# Identification of Relevant Conformational Epitopes on the HER2 Oncoprotein by Using Large Fragment Phage Display (LFPD)

**DOI:** 10.1371/journal.pone.0058358

**Published:** 2013-03-28

**Authors:** Federico Gabrielli, Roberto Salvi, Chiara Garulli, Cristina Kalogris, Serena Arima, Luca Tardella, Paolo Monaci, Serenella M. Pupa, Elda Tagliabue, Maura Montani, Elena Quaglino, Lorenzo Stramucci, Claudia Curcio, Cristina Marchini, Augusto Amici

**Affiliations:** 1 Department of Bioscience and Biotechnology, University of Camerino, Camerino (MC), Italy; 2 Faculté de Médecine, Biothérapie du Diabète, Université de Lille 2, Lille, France; 3 Department of Methods and Models for Economics, Territory and Finance, Sapienza University of Rome, Roma, Italy; 4 Department of Statistical Science, Sapienza University of Rome, Roma, Italy; 5 Molecular Targeting Unit, Department of Experimental Oncology and Molecular Medicine, Fondazione IRCCS, Istituto Nazionale dei Tumori, AmadeoLab, Milan, Italy; 6 Molecular Biotechnology Center, University of Torino, Torino, Italy; 7 Aging Research Centre, G. d'Annunzio University, Chieti, Italy; VTT Technical Research Centre of Finland and University of Turku, Finland

## Abstract

We developed a new phage-display based approach, the Large Fragment Phage Display (LFPD), that can be used for mapping conformational epitopes on target molecules of immunological interest. LFPD uses a simplified and more effective phage-display approach in which only a limited set of larger fragments (about 100 aa in length) are expressed on the phage surface. Using the human HER2 oncoprotein as a target, we identified novel B-cell conformational epitopes. The same homologous epitopes were also detected in rat HER2 and all corresponded to the epitopes predicted by computational analysis (PEPITO software), showing that LFPD gives reproducible and accurate results. Interestingly, these newly identified HER2 epitopes seem to be crucial for an effective immune response against HER2-overexpressing breast cancers and might help discriminating between metastatic breast cancer and early breast cancer patients. Overall, the results obtained in this study demonstrated the utility of LFPD and its potential application to the detection of conformational epitopes on many other molecules of interest, as well as, the development of new and potentially more effective B-cell conformational epitopes based vaccines.

## Introduction

HER2, also known as ErbB2 or neu in rat, is a 185-kd transmembrane receptor with tyrosine kinase activity and initially identified in a rat glioblastoma model. HER2 belongs to the epidermal growth factor receptor family and consists of an extracellular binding domain, a single transmembrane-spanning domain, and a long cytoplasmic tyrosine kinase domain [1].

HER signaling network normally governs cellular programs during development and post-natal life, but its deregulation is directly involved in the pathogenesis of several human tumors [2]. Amplification and/or overexpression of HER2 have a causal role in the promotion of carcinogenesis and have been reported in several types of human carcinomas, especially in breast and ovary tumors [3]. The crucial role of HER2 in epithelial transformation as well as its selective overexpression in cancer tissues makes it an ideal target for cancer immunotherapy, such as passive immunotherapy with the humanized monoclonal antibody Trastuzumab [4]. Notwithstanding the clinically approved use of Trastuzumab, a number of concerns, including resistance, considerable costs associated with repeated treatments, and side effects, make active immunotherapies that generate polyclonal and long-lasting immune responses desirable alternative approaches. Moreover, because spontaneous anti-HER2 antibodies and T cells are detected in breast cancer patients [5], HER2 protein is expected to be an excellent target for therapeutic vaccines against HER2-overexpressing cancers [6]. An abundance of experiments in preclinical models demonstrates the promise of DNA vaccination as an effective approach to prevent the development of HER2-positive tumors, eliciting immune protection against spontaneous mammary carcinomas in mice transgenic for the rat HER2 oncogene as well as in transplantable rat and human HER2-expressing tumors [7]–[13]. Anti-HER2 antibody production after vaccination represents the main mechanism responsible for the anti-tumor response [11]. In fact, anti-HER2 antibodies are able to downmodulate the expression of this growth factor receptor causally implicated in carcinogenesis. Indirect reactions, such as antibody dependent cellular cytotoxicity (ADCC) and complement-mediated cytotoxicity, are also crucial in preventing the onset of a tumor and controlling its progression.

However, the promising results obtained in preclinical models are difficult to reproduce in advanced cancers, when the immune system is already severely weakened. The generation of an effective vaccine able to trigger a long-lasting immunity that prevents tumor recurrence in cancer patients implies the understanding of how tolerance, immunity and immunosuppression regulate antitumor immune responses. Equally important for the rational design of cancer vaccines is the development of new biotechnological tools for the identification of the most immunogenic portions of a molecule and for the selection of the key epitopes within a protein.

Despite the crucial relevance of conformational B-cell epitopes, both for diagnostic and immunotherapeutical applications, methods for their detection are still lacking. For this reason we have generated a new technique, based on phage display library approach, that permits to express and displays on the phage surface protein fragments that fold reproducing native conformational epitopes.

## Results and Discussion

**Figure 1:**
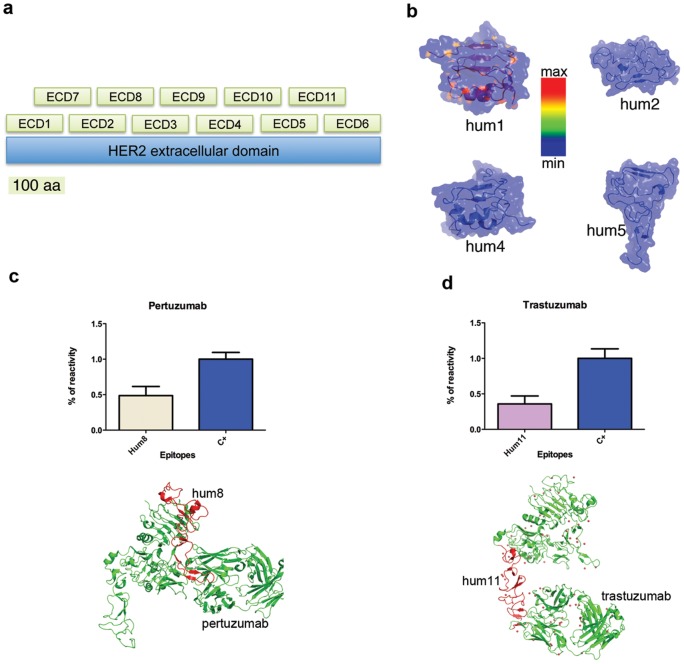
Conformational epitope mapping by LFPD. LFPD structure (a). The extracellular domain of rat and human HER2 was divided into 11 fragments (respectively of 106 and 109 amino acids): 6 contiguous core fragments plus 5 fragments overlapping the previous ones. Homology modeling of conformational epitopes (b). Molecular modeling analysis showed that the folding of each single fragment resembles the folding of the respective fragment in the entire molecule (blue zones). Only structures of hum1-2-4-5 fragments are shown for simplicity. Epitope mapping with Pertuzumab (c). Pertuzumab recognized only hum8 fragment on LFPD. Schematic representation of crystal structure of Pertuzumab (green)/hum8 fragment (red) complex (c, below). Epitope mapping with Trastuzumab (d). Trastuzumab recognized only hum11 fragment on LFPD (d). Schematic representation of cristal structure of Trastuzumab (green)/hum11 fragment (red) complex (d, below). Whole ECD of human HER2 was used as positive control (C+) (c, d).

**Figure 2:**
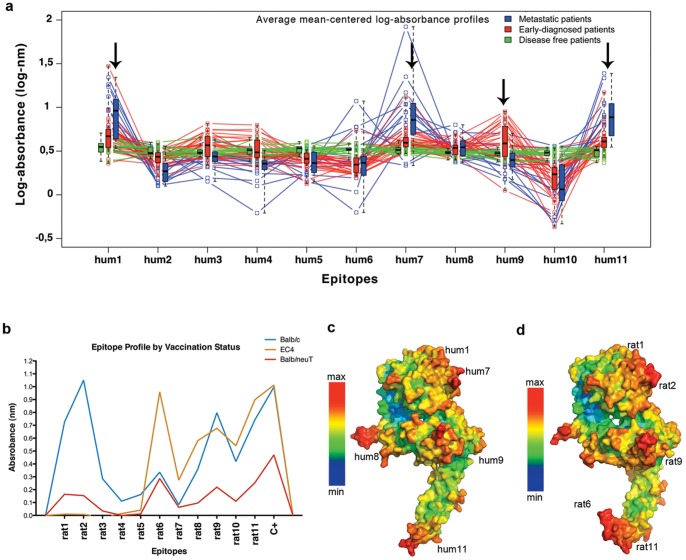
Identification of relevant conformational epitopes on HER2 oncoprotein. Screening of sera from metastatic and early breast cancer patients using an ELISA assay based on human LFPD (a). Sera from metastatic breast cancer patients recognized hum1, hum7, and hum11 fragments (blue lines). Sera from early breast cancer patients recognized hum1, hum7, hum9 and hum11 fragments (red lines). Green lines represent sera from healthy donor patients. Screening of sera from mice vaccinated with EC-TM plasmid by an ELISA assay based on rat LFPD (b). Both sera from non-tolerant Balb/c (blue line) and tolerant Balb/neuT (red line) mice recognized rat1, rat2, rat9 and rat11 conformational epitopes, although tolerant mice sera showed a lower reactivity. Sera from mice vaccinated with a truncated form of HER2 (EC4) recognized rat6, rat9 and rat11 fragments (orange line). Pepito based analysis of human HER2 (c). Pepito based analysis of rat HER2 (d). Red zones indicate the maximum probability to find a conformational epitope.

The majority of B-cell epitopes are conformational and play a critical role in the immune response [14], [15]. These epitopes typically consist of 15–20 amino acidic residues, derived from two or more discontinuous segments of a peptide that are brought together by the protein folding process, to produce the contiguous tridimensional (3D) surface recognized by the antibody [14]. Conformational epitopes are likely to induce a synergistic and more effective immunological response as compared to peptides containing linear epitopes, which are composed of a single stretch of residues [15]. Phage display of random peptide libraries is a powerful technique that has been used with some success in epitope mapping studies [16]. However, this approach is not appropriate for mapping conformational epitopes, due to their structural complexity [14], [15], [17]. To overcome this drawback, we have developed the LFPD, as a new phage display-based strategy. Contrary to the standard phage-display approach which is based on the random expression of a large number of short peptides (6–15 aa in length), in LFPD only a limited set of larger fragments (about 100 aa in length) are expressed on the phage surface. There are two sets of fragments: core and overlapping (Figure 1a). The core fragments are designed to be contiguously distributed to cover the entire peptide sequence (ECD1-ECD6). The overlapping fragments are added to allow the representation of putative conformational epitopes that could be present at the boundaries of the core fragments (ECD7-ECD11). We used this new molecular strategy, the LFPD, to reproduce the conformational epitopes of HER2. In particular, the extracellular domains of human and rat HER2 sequences (hHER2-ECD and rHER2-ECD, respectively) were considered. To generate the human LFPD, 11 fragments were expressed on the surface of phage M13 (Figure 1a). In details, the six core fragments were: hum1 (1–109 aa/1–321 bp), hum2 (110–219 aa/322–643 bp), hum3 (220–329 aa/644–965 bp), hum4 (330–439 aa/966–1.287 bp), hum5 (440–549 aa/1.288–1609 bp), hum6 (550–659 aa/1.610–1.931 bp). The five overlapping fragments were: hum7 (50–159 aa/150–471 bp), hum8 (160–269aa/472–793 bp), hum9 (270–379 aa/794–1.150 bp), hum10 (380–489 aa/1.151–1.472 bp), hum11 (490–599 aa/1.473–1.794 bp). The overlapping fragments were positioned to create an overlap of about 50 aa with respect to the core fragments. The fragments were expressed as in frame fusion to the N-terminal major g3p coat protein of filamentous phage M13 (Figure S1). As negative control we used the empty expression vector PIF6 phagemid [18]. Molecular 3D modeling was used to verify the folding of each fragment. This analysis showed that each isolated fragment retained the native folding which is present in its corresponding part of the entire molecule (Figure 1b). We next checked if the fragments were properly expressed on the phage surface. Two monoclonal antibodies, Pertuzumab and Trastuzumab, which are known to recognize two hHER2-ECD conformational epitopes [1], [19] were used for this purpose. Those epitopes are contained in hum8 (Pertuzumab) and hum11 (Trastuzumab) fragments. As expected, Pertuzumab recognized only hum8 fragment (Figure 1c) and Trastuzumab only hum11 fragment (Figure 1d), therefore, further indicating the expression of properly folded hHER2-ECD fragments on the phage surface. Then, we screened the human LFPD clones with 19 sera derived from HER2-positive metastatic breast cancer patients (MBC) and 28 sera derived from HER2-positive early breast cancer patients (EBC). Sera from healthy donors were used as negative control (HD). A preliminary flow-cytometry analysis verified that all the sera contained antibodies recognizing hHER2-ECD (Figure S2). Thereafter, all the sera were used in an ELISA assay, based on LFPD, to identify the hHER2-ECD reactive epitopes. Experimental quantitative outcomes were measured in terms of absorbance expressed in nm. Due to individual heterogeneity, the original raw outcome needed to be properly normalized in order to get appropriate comparative evidence of epitope binding. There is limited literature addressing the issue of appropriate normalization of binding signals and no universal agreement on the most appropriate technique to be used [20], [21]. Hence three alternative signal normalization techniques were investigated in order to keep under control different sources of variability which yield confounding wild fluctuations in the original raw data. Normalization as described in Figure S7 and S8 takes explicitly into account the underlying background standardizing with an appropriate function of absorbance increment in the two control spots (Negative and Positive, see details in the corresponding captions). Figure S9 shows the data normalized using the mean centering of the logarithmic transformation of the original raw absorbance: this transformation takes into account some background implicitly by averaging the absorbance over all fragments. This third choice has been preferred to better discriminate among the HD, EBC and MBC groups (see Figure S15 and S16). Indeed, Wilcoxon and T test, when applied separately to each version of the normalized data, select the same list of significant binding epitopes. Wilcoxon unidirectional tests (significance level α = 0.05) were used to single out overall epitope binding for each fragment. Significant statistical binding was detected for hum1 (p-value<0.001), hum7 (p-value<0.001) and hum11 (p-value<0.001) for the metastatic patients (MBC) and hum1 (p-value<0.001), hum7 (p-value<0.001), hum9 (p-value = 0.025), hum11 (p-value<0.001) for the early group. Alternative normalization techniques were used and provided similar results (see Figures S3, S4, S5, S6, S7, S8, S9, S10, S11, S12, S13, S14, S15, and S16 and Methods S1 for details). The ordering of the absorbance outcome normalized via mean centering on the log scale revealed also some interesting potential for group discrimination, as it can be realized from the first two principal component mapping in Figure S15 and from the dendrogram in Figure S16 (see Methods S1 for further details), MBC, EBC and HD patients are clear-cut clustered. The sera from MBC recognized 3 epitopes on fragments hum1, hum7 and hum11 (Figure 2a). Interestingly, the sera from EBC recognized the same 3 epitopes recognized by sera from MBC (hum1, hum7 and hum11), plus an additional epitope, positioned on hum9 fragment (Figure 2a). The possibility to use the epitope contained in fragment 9 for discriminating between MBC and EBC could be of clinical interest and warrants further analysis. Finally, we used the PEPITO software analysis for the prediction of conformational epitopes [22] on the entire hHER2-ECD molecules (Figure 2c). Remarkably, the PEPITO analysis predicted the presence of the same epitopes identified by ELISA screening of breast cancer patients. In particular, PEPITO predicted conformational epitopes corresponding to the four epitopes present on fragments hum1, hum7, hum9 and hum11 of the human LFDP, therefore validating LFDP method.

To assess the reproducibility of the LFPD strategy, we generated a rat LFPD using the same design strategy used for the human LFPD. The rat LFPD was screened using the sera obtained from rat HER2 non tolerant (Balb/c) and tolerant (Balb/neuT) mice [23] immunized with a plasmid encoding the extracellular and transmembrane domains of the rat HER2 (EC-TM) [11], [12]. In wild-type Balb/c mice, rat HER2 is a foreign, xenogeneic antigen differing in less than 6% of amino acids from mouse HER2 [20]. In these mice EC-TM plasmid vaccination triggers a strong immune response. In cancer-prone rat HER2 transgenic Balb-neuT mice, rat HER2 is expressed in the thymus and induces the deletion of T cells that recognize dominant epitopes with high affinity [23]. Even under these conditions EC-TM plasmid electroporation leads to a significant delay in the progression of HER2-positive mammary lesions [10]. The screening with sera of non-tolerant mice permitted to identify conformational epitopes corresponding to fragments rat1, rat2, rat9 and rat11. This result was confirmed using the sera obtained from tolerant mice, although with a lower reactivity. In addition, sera from mice vaccinated with a truncated form of rat HER2, encoding the TM domain associated with amino acids 311 to 654 of the EC domain (EC4) [11], showed a strong reactivity for fragment rat6, other than for rat9 and rat11, and, as expected, ignored the first part of the molecule (Figure 2b). The strong reactivity toward rat6 may be explained by an immunodominant effect caused by epitopes that could be present in the first part of the molecule. Furthermore, all the conformational epitopes recognized by the polyclonal antibodies present in the sera of vaccinated mice corresponded to the epitopes predicted by PEPITO software analysis (Figure 2d).

**Figure 3:**
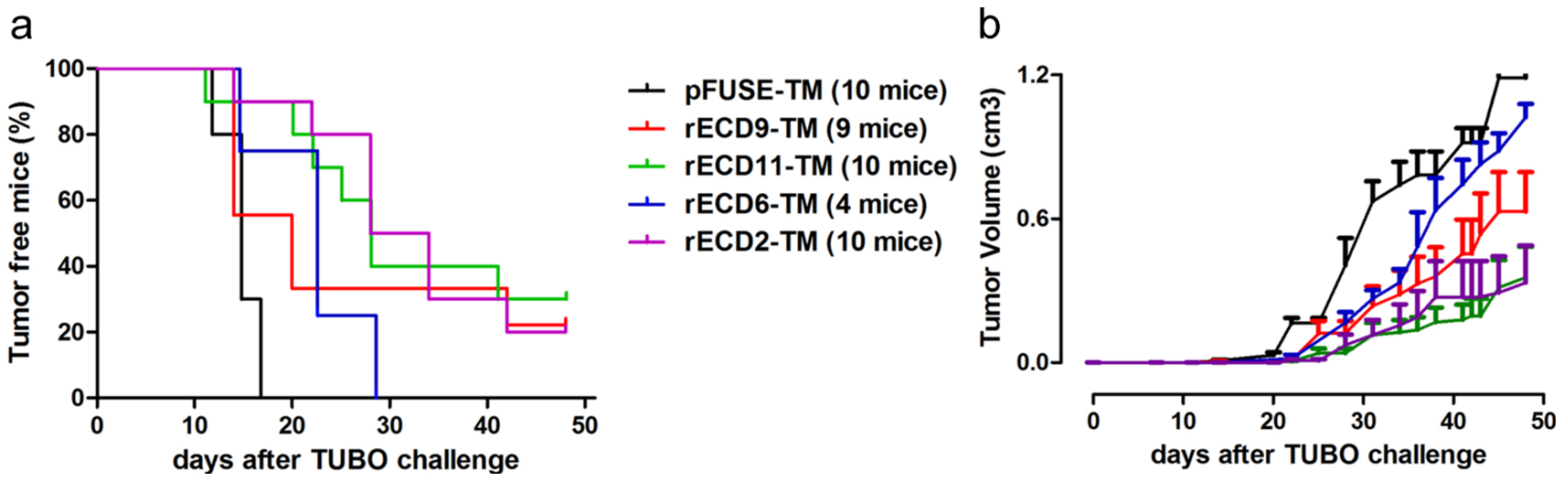
Immunogenicity of epitope-based DNA vaccines in wild-type Balb/c mice. The antitumor protection afforded by each plasmid, electroporated 21 and 7 days before a lethal rat HER2-positive TUBO cell challenge, is shown both as tumor-free survival (a) and tumor size (b).

A further evidence of the ability of LFPD to reproduce B-cell epitopes was provided by preliminary vaccination experiments. In order to selectively induce an immune response against rat2, rat6, rat9 and rat11 HER2 epitopes, we developed DNA vaccines coding for fusion proteins between the murine Fc-region of an IgG2a molecule, that acts as molecular support for the correct protein folding, and each single rat HER2 epitope (rECD2-TM, rECD6-TM, rECD9-TM, rECD11-TM). Since a transmembrane domain was inserted in each DNA vaccine, the selected antigens were exposed on the cell surface. To assess the ability of these new epitope-based DNA vaccines to trigger an anti-HER2 immune response, 50 µg of DNA plasmids were electroporated into the femoral muscle of wild-type Balb/c mice, 14 and 7 days before challenge with a lethal dose of a single-cell suspension of syngeneic HER2-positive adenocarcinoma cells (TUBO cells). As shown by Kaplan-Meier plot (Figure 3a), whereas a transplanted tumor grew in all mice electroporated with the empty vector pFUSE-TM within 18 days after TUBO challenge, vaccination with epitope-based vaccines delayed significantly the tumor onset and substantial protection, ranging from 20 to 30%, was displayed by mice electroporated respectively with rECD2-TM (2/10 tumor free/total mice) or with rECD9-TM (2/9 tumor free/total mice) and rECD11-TM (3/10 tumor free/total mice), on day 50 after challenge when the experiment ended. Of note, as shown in the tumor growth graph, tumors developed in the rECD9-TM vaccinated mice were about half in volume in comparison with tumors developed in control mice, and tumors developed in rECD2-TM and rECD11-TM vaccinated mice remained very small, with an average volume of 0.3 cm^3^, until the end of the experiment (Figure 3b). A delay in tumor onset and a reduction in the tumor growth rate were found in mice electroporated with rECD6-TM plasmid, but they all eventually displayed tumors (Figure 3). Analysis of the sera from immunized mice revealed that the immunoprotection elicited by epitope-based vaccines was associated with an antibody response, demonstrating that antibodies raised against the selected epitopes can recognize the native HER2 protein (Figure S18).

In conclusion, using LFPD, we were able to identify four new conformational epitopes which were the same in both human and rat HER2. Given the relative simplicity of LFPD, we envisage the application of this new tool to the development of new vaccines that are based on the identification of B-cell conformational epitopes. In particular, preliminary results demostrate that these epitope-based vaccines are able to induce an immunoprotection against HER2-overexpressing tumors.

In addition, since LFPD might help discriminating between metastatic breast cancer and early breast cancer patients, a potential LFPD application could be also the breast cancer diagnostic analysis. Finally, another interesting field of application could be the production of patient-specific monoclonal antibodies, opening the way to a more personalized therapeutic approach in patients suffering from cancer and other diseases that can be treated with immunotherapy.

## Materials and Methods

### Phages production and purification

All the human and rat HER2 fragments were cloned in frame with g3p of M13 phage to generate two sets (one for rat and one for human sequences) of phage-displayed clones. The sequences of the resulting plasmids were verified by sequencing (BMR Genomics, Padua, Italy). TG1 cells containing DNA plasmids coding for library fragments were superinfected with helper phage K07 at m.o.i 20. Phages expressing fragments were purified with 3/10 v/v of PEG-NaCl.

### Enzyme-linked immunosorbent assay

Enzyme-linked immunosorbent assays (ELISAs) were constructed using PEG purified HER2 fragments – expressing phages isolated as described above. Ninety-six-well microtiter plates (Maxi-sorb, NUNC Rochester, NY) were coated with purified phage particles (10^12^ phage per well in PBS/BSA at 4°C overnight), blocked (PBS/BSA, 37°C×1 hr) and washed (PBS/Tween 20). Mouse or patient serum was added to individual wells (37°C×1 hr) and washed, then incubated with anti-human or anti-mouse alkaline phosphatase-conjugated secondary antibody (37°C×1 hr). Pertuzumab and Trastuzumab (Herceptin) were provided by Genentech. Empty phages were used as a negative control. Assays were developed with pNPP (p-Nitrophenyl Phosphate, Disodium Salt) substrate (Sigma). The absorbance was read spectro-photometrically at 405 nm.

### Molecular modeling and conformational epitope prediction

All the calculations have been carried out on MacPro (Apple, Cuppertino, Ca) workstations, using the Discovery Studio 2.1 (Accelrys, Inc.) software package. The rat 1-11 fragment structures were constructed with homology modeling using the extracellular portion of rat HER2 (1n8yc) as template and Swiss-Model Server with SPDBV program suite 3.0. The structures were then submitted to minimization cycles, using a Newton-Raphson minimization algorithm with at least 5000 iterations. The minimization cycles were repeated until energy global minimum was reached. The models obtained were checked using standard validation programs as PROCHECK e VERIFY_3D. The prediction of conformational epitopes has been carried out on the proteins human HER2 (1n8z) and rat HER2 (1n8y). The folding was evaluated measuring euclidean distance between homologous atoms, precisely between α-carbon using a bioinformatic tool that was developed by prof. Mauro Angeletti at University of Camerino.

### Cell lines

Balb/c 3T3 fibroblasts, stably cotransfected with rat HER2 and mouse class I H-2Kd and B7.1 genes (3T3/NKB), kindly provided by W.-Z. Wei (Karmanos Cancer Institute, Detroit, MI) [24] were cultured in Dulbecco's Modified Essential Medium (DMEM) with Glutamax I medium (Life Technologies, San Giuliano, Italy), supplemented with 10% FBS, penicillin (50U/ml)/streptomycin (50 μg/ml) and with 0.6 mg/mL G418 (geneticin, Invitrogen) and 0.6 mg/mL zeocin (Invitrogen). TUBO cells, a cloned cell line established in vitro from a lobular carcinoma that arose in a female BALB-neuT mouse [8], were cultured in DMEM with 20% FBS. Human embryonic kidney cells (HEK293), stable transfected with human HER2, were cultured in RPMI medium with 10% FBS, 1% glutamine, 1% penicillin/streptomycin and G418 antibiotic. Cells were maintained at 37°C in an atmosphere of 5% CO_2_.

### Flow cytometric analysis

Indirect immunofluorescence assay was performed on live cells (HEK293 stable transfected with human HER2) with human sera. Cells were incubated with sera (1∶200) for 30 min at 37°C, washed twice, and incubated with goat anti-human IgG (1∶100) (Kirke-gaard & Perry Laboratories, Gaithersburg, MD, USA) for 30 min at 0°C. After a final wash, cells were suspended in PBS. Fluorescence was analyzed by FACScalibur with CellQuest and FLowJo software (Becton Dickinson, Mountain View, CA, USA).

### Epitope-based DNA vaccines

pFUSE-mIgG2A-Fc2 (Invivogen, San Diego, CA) was used as backbone for the construction of epitope-based DNA vaccines. To anchor the encoded protein to the plasma membrane, a synthetic oligo codifying the TM domain of rat HER2 was cloned in frame in pFUSE-mIgG2A-Fc2 using NheI restriction enzyme (Celbio, Milan, Italy). The cDNA fragments codifing for rat2, rat6, rat9 and rat11, were obtained by PCR using pVax-ECTM [12] as template and the following primers: 5′GCGCGAATCCGCCTCCACCCCAGGCAGAACC3′ (rat2 sense); 5′GCGCCCATGGGGGCAGCCGGCCCTTGCACCG3′ (rat2 antisense); 5′GCGCGAATTCCCTCCCCCGGGAGTATGTGAGT3′ (rat6 sense); 5′GCGCCCATGGGAATGTCACCGGGCTGGCTCT3′ (rat6 antisense); 5′GCGCGAATCCGAGGGTCGCTACACCTTTGGT3′ (rat9 sense); 5′GCGCCCATGGCGGAGCAATGCCGGAGGAGGG3′ (rat9 antisense); 5′GCGCGAATCCAACCGGCCGGAAGAGGACTTG3′ (rat11 sense); 5′GCGCCCATGGCGGAGCAATGCCGGAGGAGGG 3′ (rat11 antisense).

After purification and digestion with EcoRI and NcoI restriction enzymes (Celbio, Milan, Italy), the amplified fragments were inserted in pFUSE-mIgG2A-Fc2 with HER2 TM. rEC2-TM, rEC6-TM, rEC9-TM and rEC11-TM sequences were verified by sequencing (BMR Genomics, Padua Italy).

### DNA immunization

DNA vaccination was performed on female wild-type Balb/c mice through two cycles of DNA injection followed by electroporation, at 21 and 7 days before tumor challenge. Briefly, after i.m. injection of 50 μg of DNA plasmid, two low voltage pulses of 150 V of 25 ms with a 300 μs interval were applied through the insertion of Cliniporator needles (Igea, Carpi, Italy) into the mouse quadriceps muscles. Mice were challenged s.c. in the left flank with 0.2 ml of a single suspension containing the minimal lethal dose of TUBO cells (1×10^5^). Transplanted tumor growth was monitored weekly by palpation. Progressively growing masses >1 mm mean diameter were regarded as tumors. Mice were treated according to the European Community guidelines. The Animal Research Committee of the University of Camerino authorized the experimental protocol.

To evaluate the antibody response, sera were collected from mice the day before the TUBO challenge and the presence of anti-rat HER2 antibodies was determined by an ELISA assay using 3T3/NKB cells. Briefly, cells were seeded in a 96-well plate at a cell density of 2×10^4^ cells/well. Thirty-six hours later, cells were fixed with PBS-1% paraformaldehyde, blocked with PBS-10% BSA and incubated with sera (1∶50) from immunized animals (37°C×1 hr). Sera from mice immunized with the pFUSE-TM empty palsmid were used as a negative control. Peroxidase-conjugated goat anti-mouse IgG (1∶3000; Calbiochem) was used (37°C×1 hr) to detect bound antibodies. The assay was developed by adding 2,2-azino-bis-3 ethylbenzothiazoline-6-sulfonic acid as enzymatic substrate (Sigma). The absorbance was read spectro-photometrically at 405 nm wavelength.

## Supporting Information

Figure S1 **Molecular structure of a rat HER2 fragment fused with g3p of M13 phage.** Rat1 (blu)-g3p (orange) structure was shown as representative image. It was generated and were minimized until energy global minimum was reached. Structures were constructed with homology modeling using the extracellular portion of rat HER2 (1n8yc) as template and Swiss-Model Server with SPDBV program suite 3.0. The structures were then submitted to minimization cycles, until energy global minimum was reached.
(TIF)

Figure S2 **Detection of antibodies against human HER2 extracellular domain in human sera.** Flow cytometric analysis shows the reactivity of representative sera (blue lines), from two different metastatic patients (22OM on the left and 2OM on the right), with HEK293 cells, stable transfected with human HER2. Anti-HER2 serum antibodies were detected using a FITC-conjugated goat anti-human IgG. Trastuzumab (10 μg/ml) was used as positive control (green lines). The red lines indicate the background values. The x axis represents fluorescence intensity, and the y-axis represents relative cell number.
(TIF)

Figure S3 **Human LFPD.** Raw absorbance of 60 ELISA experiments (triplicated) on human sera. Absorbance profile of 13 wells corresponding to 11 fragments (hum1-hum11) and 2 control wells (CtrlPos, positive control  =  whole HER2 protein; CtrlNeg, negative control  =  phage). Results are displayed by health status group: disease free (green), early-diagnosed cancer (red), metastatic (blue).
(TIF)

Figure S4 **Human LFPD.** Log-transformed raw absorbance of 60 ELISA experiments (triplicated) on human sera. Absorbance profile of 13 wells corresponding to 11 fragments (hum1-hum11) and 2 control wells (CtrlPos, positive control  = whole HER2 protein; CtrlNeg, negative control  =  phage). Results are displayed by health status group: disease free (green), early-diagnosed cancer (red), metastatic (blue).
(TIF)

Figure S5 **Human LFPD.** Mean-centering normalization: log-absorbances of 60 ELISA experiments (triplicated) on human sera have been normalized by subtracting log-absorbance mean of each single replicate profile. Normalized profile of 13 wells displayed by health status group: disease free (green), early-diagnosed cancer (red), metastatic (blue).
(TIF)

Figure S6 **Human LFPD.** Mean-centering normalization of triplicates: log-absorbances of 60 ELISA experiments on human sera have been normalized by subtracting log-absorbance mean of each triplicate profile. Normalized profile of 13 wells displayed by health status group: disease free (green), early-diagnosed cancer (red), metastatic (blue).
(TIF)

Figure S7 **Human LFPD.** Relative increment normalization of triplicates: raw absorbances of 60 ELISA experiments on human sera have been normalized by dividing each by the absorbance increment in the two control spots of each replicate. Normalized profile of 13 wells displayed by health status group: disease free (green), early-diagnosed cancer (red), metastatic (blue).
(TIF)

Figure S8 **Human LFPD.** Log-absorbance exceedance normalization of triplicates: log-absorbances of 60 ELISA experiments on human sera have been normalized by subtracting a 25% of the log-absorbance increment in the two control spots of each replicate. Normalized profile of 13 wells displayed by health status group: disease free (green), early-diagnosed cancer (red), metastatic (blue). Letters “W” (“T”) those epitopes for which a significant binding has been detected (p-value<0.05) with the Wilcoxon test (Student T test, whenever appropriate).
(TIF)

Figure S9 **Human LFPD.** Mean-centering normalization of triplicates. Same data as in Figure S4 with superimposed boxplots for each fragment to highlight summary characteristics of the overall profile shape.
(TIF)

Figure S10 **Rat LFPD.** Relative increment normalized absorbance of 8 ELISA experiments (triplicated) on rat sera. Absorbance profile of 11 wells corresponding to 11 fragments (rat1-rat11). Results are displayed by mice type: EC4-TM vaccinated Balb/c (dark-green), EC-TM vaccinated tolerant transgenic BALB-neuT (orange), EC-TM vaccinated non tolerant wild-type Balb/c (cyan).
(TIF)

Figure S11 **Rat LFPD.** Mean centered normalized log-absorbance of 8 ELISA experiments (triplicated) on rat sera. Absorbance profile of 11 wells corresponding to 11 fragments (rat1-rat11). Results are displayed by mice type: EC4-TM vaccinated Balb/c (dark-green), EC-TM vaccinated tolerant transgenic BALB-neuT (orange), EC-TM vaccinated non tolerant wild-type Balb/c (cyan).
(TIF)

Figure S12 **Rat LFPD.** Log-absorbance exceedance normalization of 8 ELISA experiments (triplicated) on rat sera. Absorbance profile of 11 wells corresponding to 11 fragments (rat1-rat11). Results are displayed by mice type: EC4-TM vaccinated Balb/c (dark-green), EC-TM vaccinated tolerant transgenic BALB-neuT (orange), EC-TM vaccinated non tolerant wild-type Balb/c (cyan).
(TIF)

Figure S13 **Human LFPD.** Pertuzumab monoclonal antibody absorbance by alternative normalizations of triplicates: normalized absorbances of 3 ELISA experiments. Normalized profiles of 3 wells corresponding to fragments (hum3, hum8, hum9) displayed by normalization type.
(TIF)

Figure S14 **Human LFPD.** Trastuzumab monoclonal antibody absorbance by alternative normalizations of triplicates: normalized absorbances of 3 ELISA experiments. Normalized profiles of 2 wells corresponding to fragments (hum6 and hum11) displayed by normalization type.
(TIF)

Figure S15 **Human LFPD.** Biplot of the first two principal components extracted from combining original normalized profiles and their ranks.
(TIF)

Figure S16 **Human LFPD.** Unsupervisioned hierarchical clustering obtained from mean centered log-absorbance. Inpt data were based on the first 6 principal components extracted from combining original normalized profiles and their ranks.
(TIF)

Figure S17 **Raw absorbance of ELISA experiments (triplicated) on human sera of two groups of cancer patients: treated with Trastuzumab (light blue) not treated with Trastuzumab (dark blue).** Absorbance profile of 13 wells corresponding to 11 fragments (hum1-hum11) and 2 control wells (CtrlPos, positive control  =  whole HER-b protein; CtrlNeg, negative control  =  phage).
(TIF)

Figure S18 **Induction of anti-HER2 antibody response by epitope-based vaccines.** Balb/c mice were vaccinated with rECD2-TM, rECD6-TM, rECD9-TM, rECD11-TM. Binding of rat HER2 expressing 3T3/NKB cells with immune sera was measured by ELISA assay as described in Materials and Methods. Sera from mice vaccinated with pFuse-TM empty plasmid were used as negative control. Results are expressed as absorbance (optical density at 405 nm). Data are shown as mean ± SEM (n = 4).
(TIF)

Table S1 **Fragment binding significance analysis for relative increment raw absorbance data OM group (metastatic breast cancer patients).**
(TIF)

Table S2 **Fragment binding significance analysis for relative increment raw absorbance data EARLY group (early breast cancer patients).**
(TIF)

Table S3 **Counts of threshold exceedance for centered log-absorbance data.**
(TIF)

Table S4 **Counts of threshold exceedance for benchmarked log-absorbance data.**
(TIF)

Methods S1 **Statistical analysis, including data transformation and normalization, testing significant overall epitope recognition, highlighting individual epitope recognition, hints of group discrimination by profile pattern.**
(DOC)
