# Supplementary figures and images for: Identification of Relevant Conformational Epitopes on the HER2 Oncoprotein by Using Large Fragment Phage Display (LFPD)
Source: PLoS One. 2013 Mar 28;8(3):e58358. doi: 10.1371/journal.pone.0058358 (PMC3610777; doi:10.1371/journal.pone.0058358)

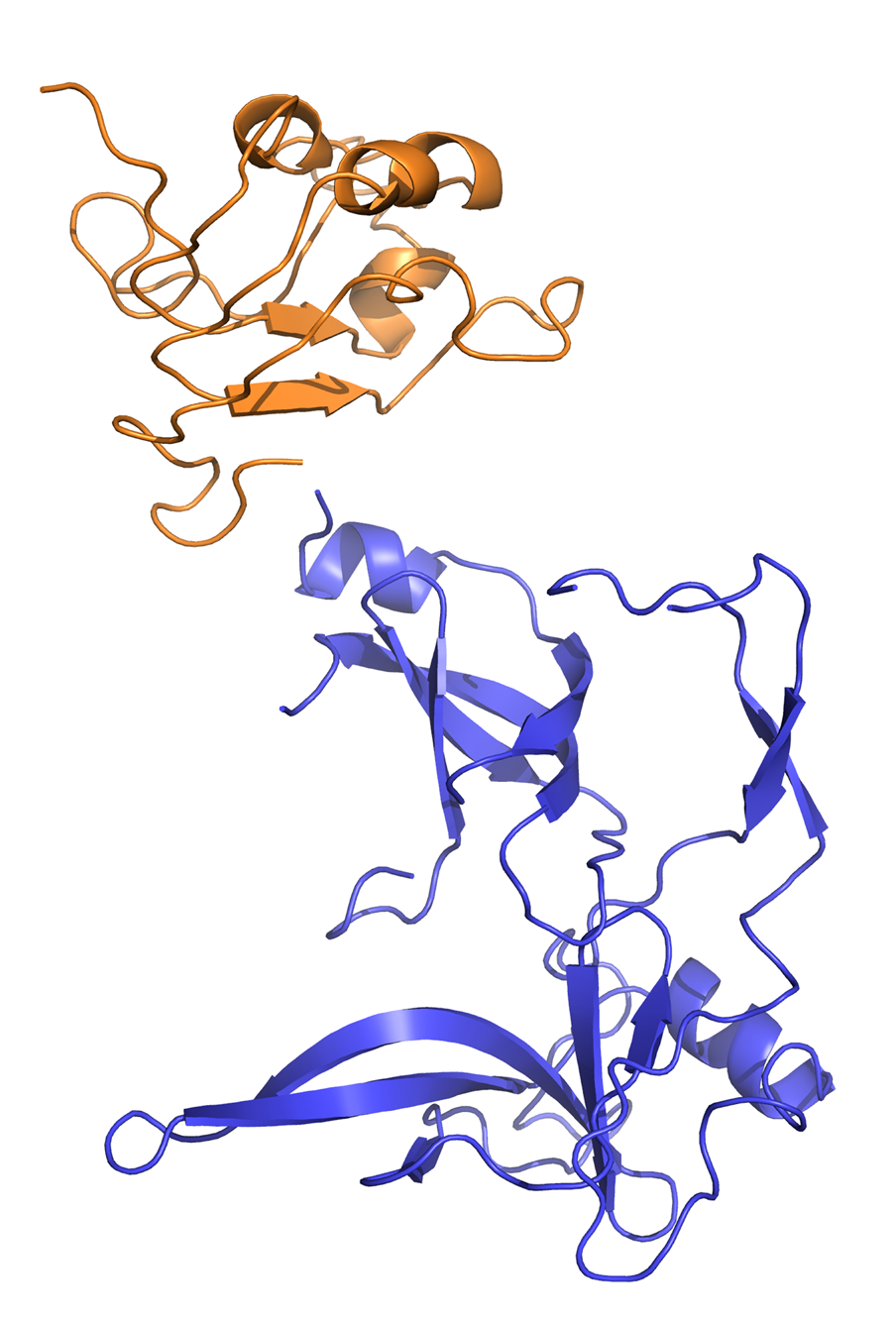

Supplement: Figure S1 — Molecular structure of a rat HER2 fragment fused with g3p of M13 phage. Rat1 (blu)-g3p (orange) structure was shown as representative image. It was generated and were minimized until energy global minimum was reached. Structures were constructed with homology modeling using the extracellular portion of rat HER2 (1n8yc) as template and Swiss-Model Server with SPDBV program suite 3.0. The structures were then submitted to minimization cycles, until energy global minimum was reached. (TIF) [file pone.0058358.s001.tif]

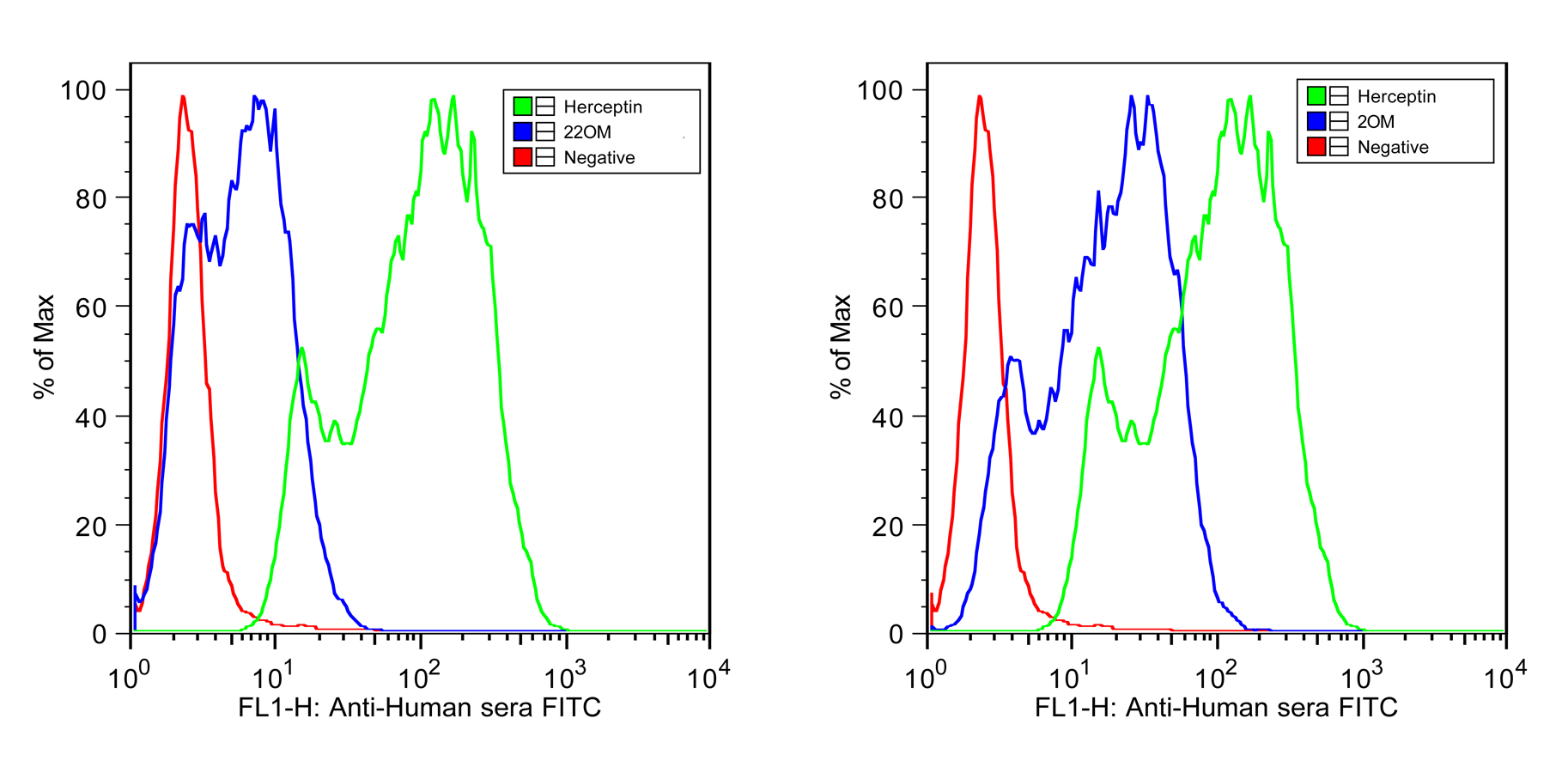

Supplement: Figure S2 — Detection of antibodies against human HER2 extracellular domain in human sera. Flow cytometric analysis shows the reactivity of representative sera (blue lines), from two different metastatic patients (22OM on the left and 2OM on the right), with HEK293 cells, stable transfected with human HER2. Anti-HER2 serum antibodies were detected using a FITC-conjugated goat anti-human IgG. Trastuzumab (10 μg/ml) was used as positive control (green lines). The red lines indicate the background values. The x axis represents fluorescence intensity, and the y-axis represents relative cell number. (TIF) [file pone.0058358.s002.tif]

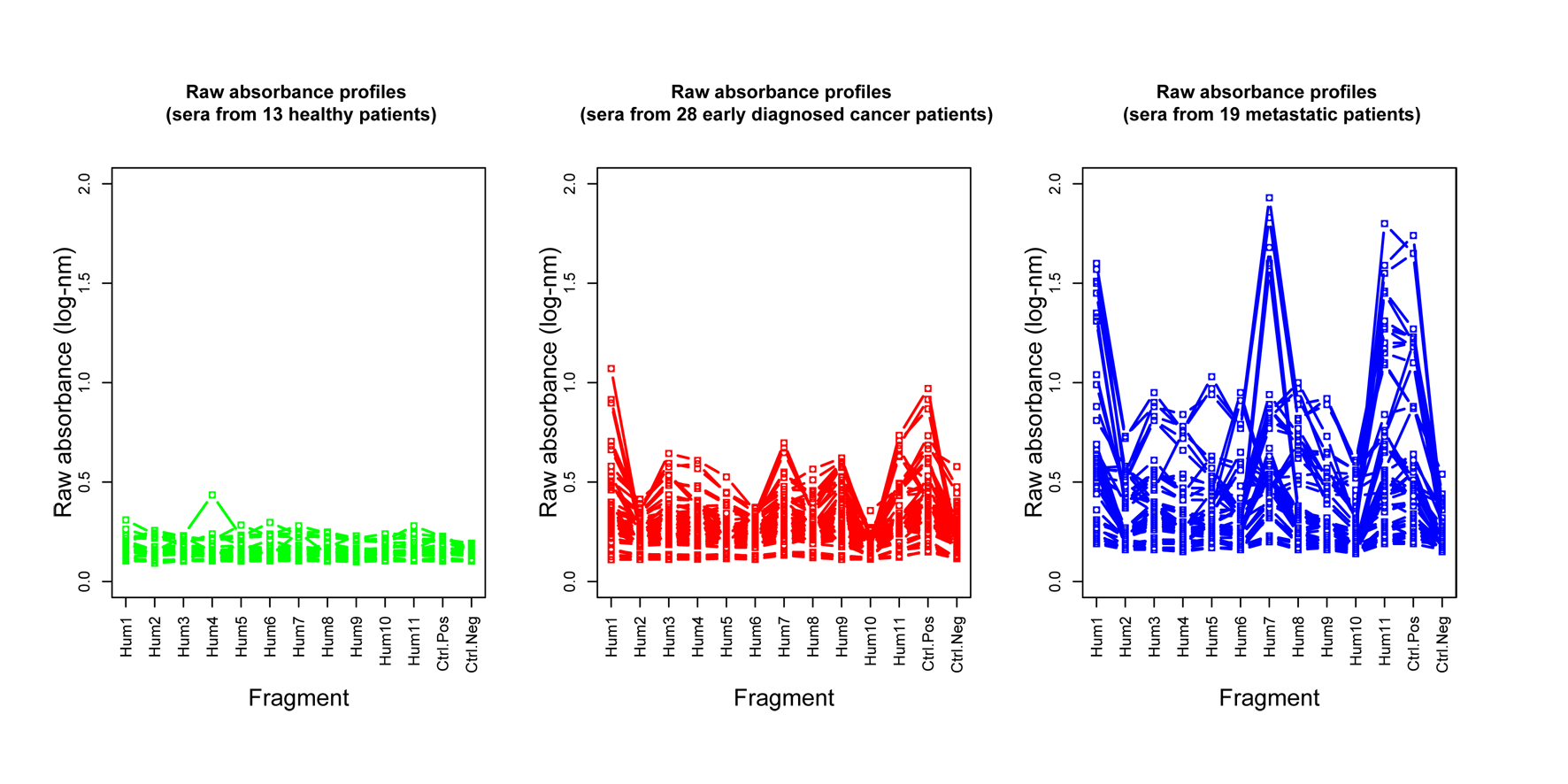

Supplement: Figure S3 — Human LFPD. Raw absorbance of 60 ELISA experiments (triplicated) on human sera. Absorbance profile of 13 wells corresponding to 11 fragments (hum1-hum11) and 2 control wells (CtrlPos, positive control = whole HER2 protein; CtrlNeg, negative control = phage). Results are displayed by health status group: disease free (green), early-diagnosed cancer (red), metastatic (blue). (TIF) [file pone.0058358.s003.tif]

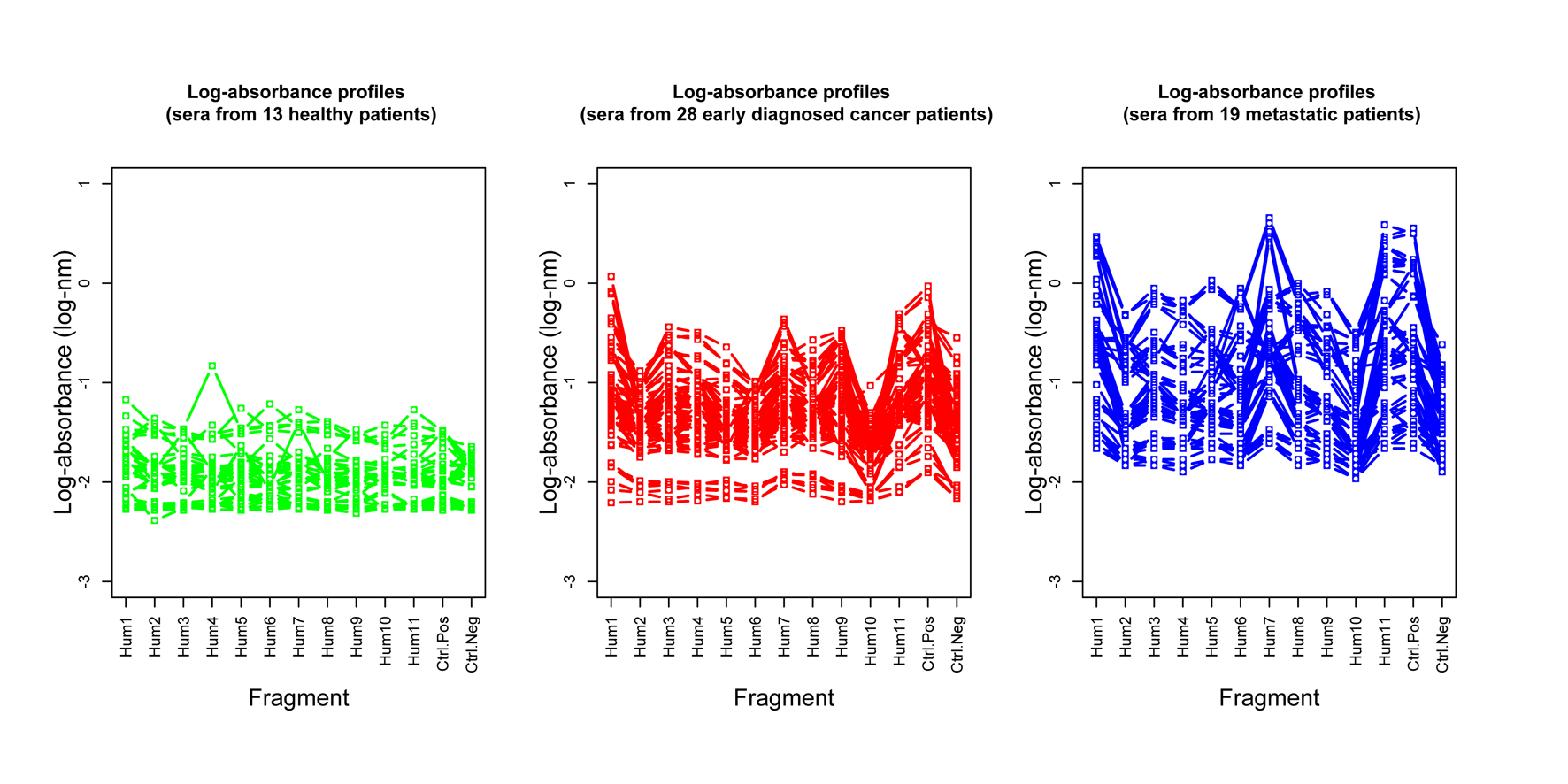

Supplement: Figure S4 — Human LFPD. Log-transformed raw absorbance of 60 ELISA experiments (triplicated) on human sera. Absorbance profile of 13 wells corresponding to 11 fragments (hum1-hum11) and 2 control wells (CtrlPos, positive control = whole HER2 protein; CtrlNeg, negative control = phage). Results are displayed by health status group: disease free (green), early-diagnosed cancer (red), metastatic (blue). (TIF) [file pone.0058358.s004.tif]

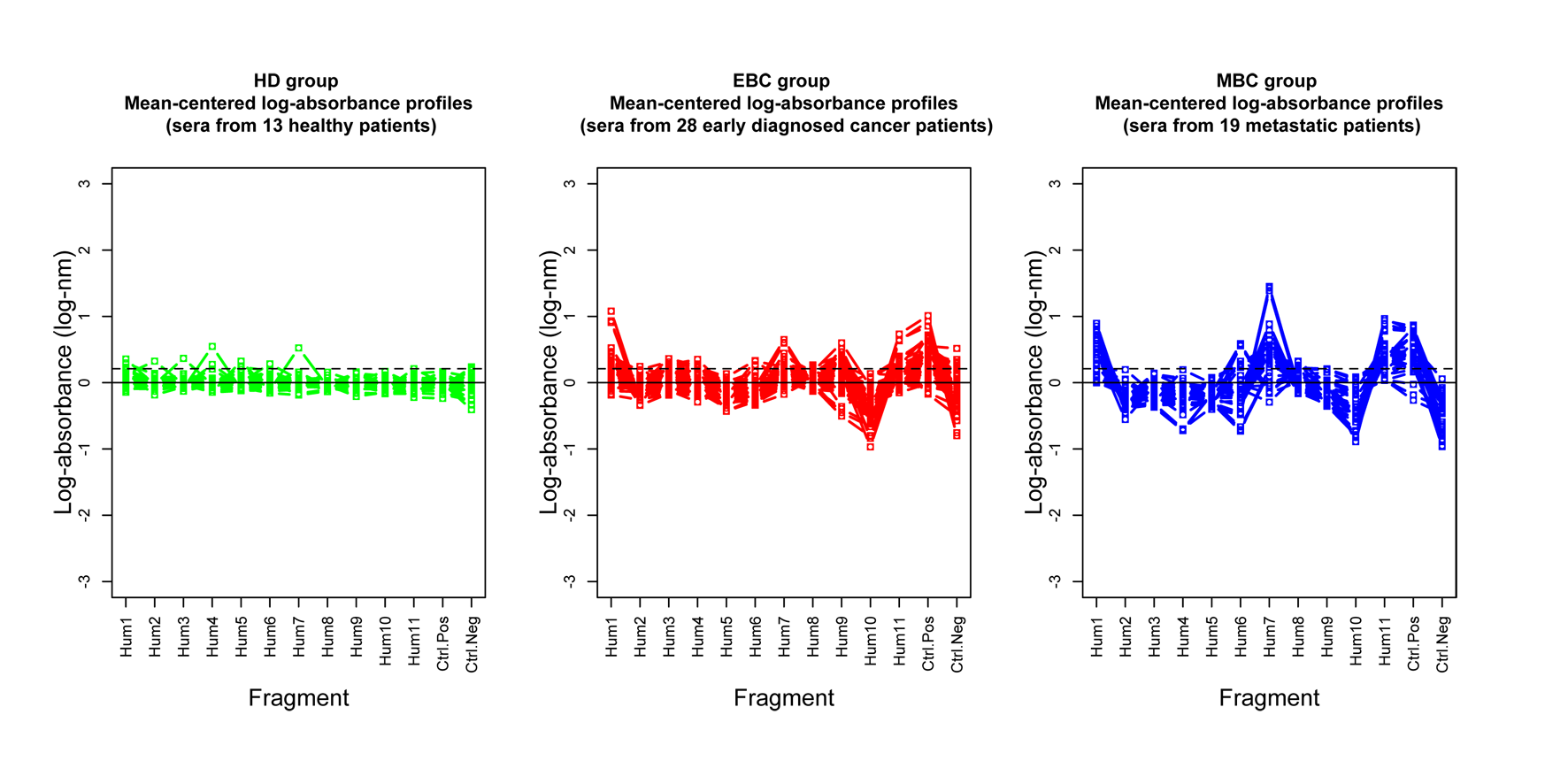

Supplement: Figure S5 — Human LFPD. Mean-centering normalization: log-absorbances of 60 ELISA experiments (triplicated) on human sera have been normalized by subtracting log-absorbance mean of each single replicate profile. Normalized profile of 13 wells displayed by health status group: disease free (green), early-diagnosed cancer (red), metastatic (blue). (TIF) [file pone.0058358.s005.tif]

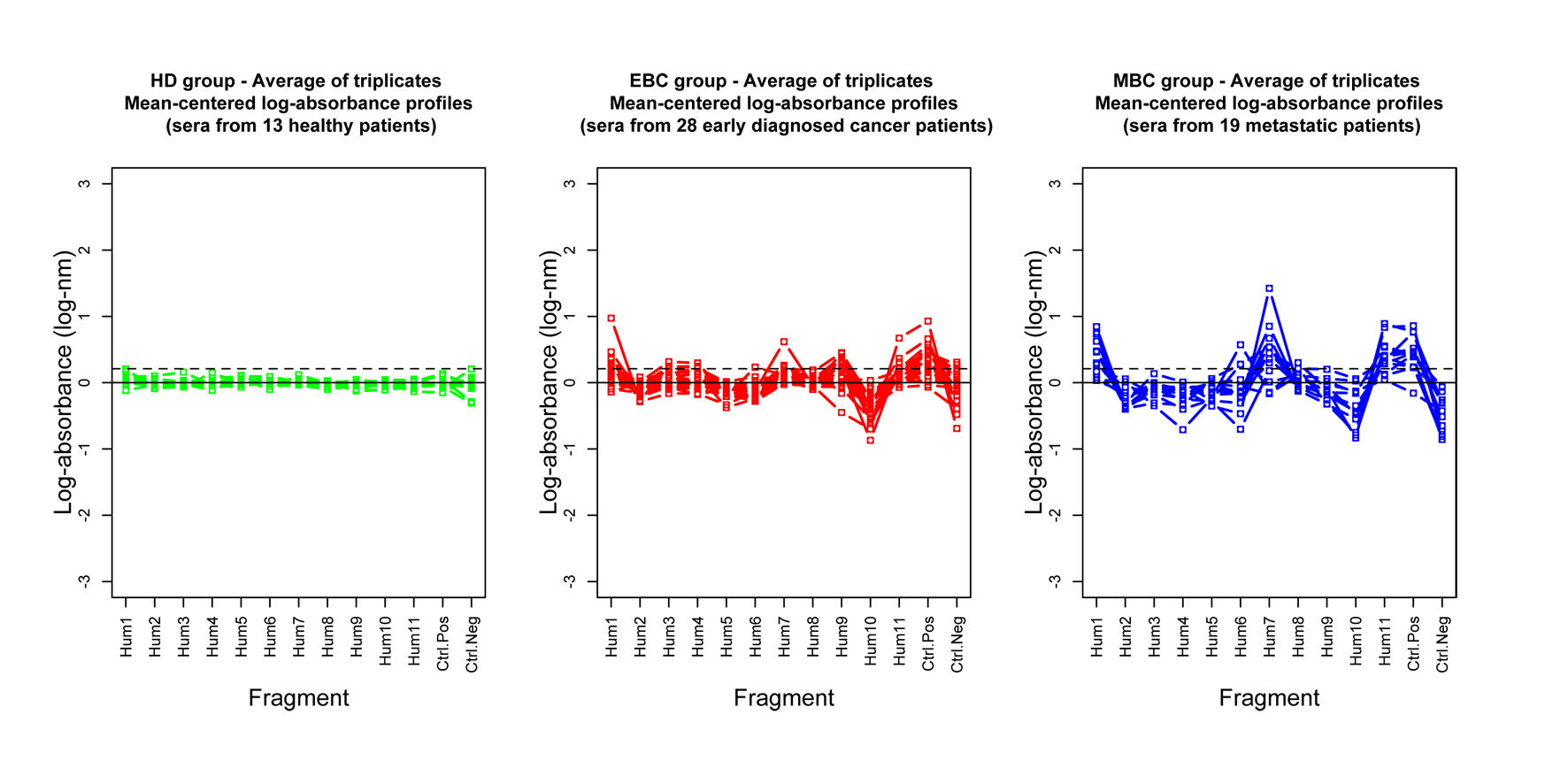

Supplement: Figure S6 — Human LFPD. Mean-centering normalization of triplicates: log-absorbances of 60 ELISA experiments on human sera have been normalized by subtracting log-absorbance mean of each triplicate profile. Normalized profile of 13 wells displayed by health status group: disease free (green), early-diagnosed cancer (red), metastatic (blue). (TIF) [file pone.0058358.s006.tif]

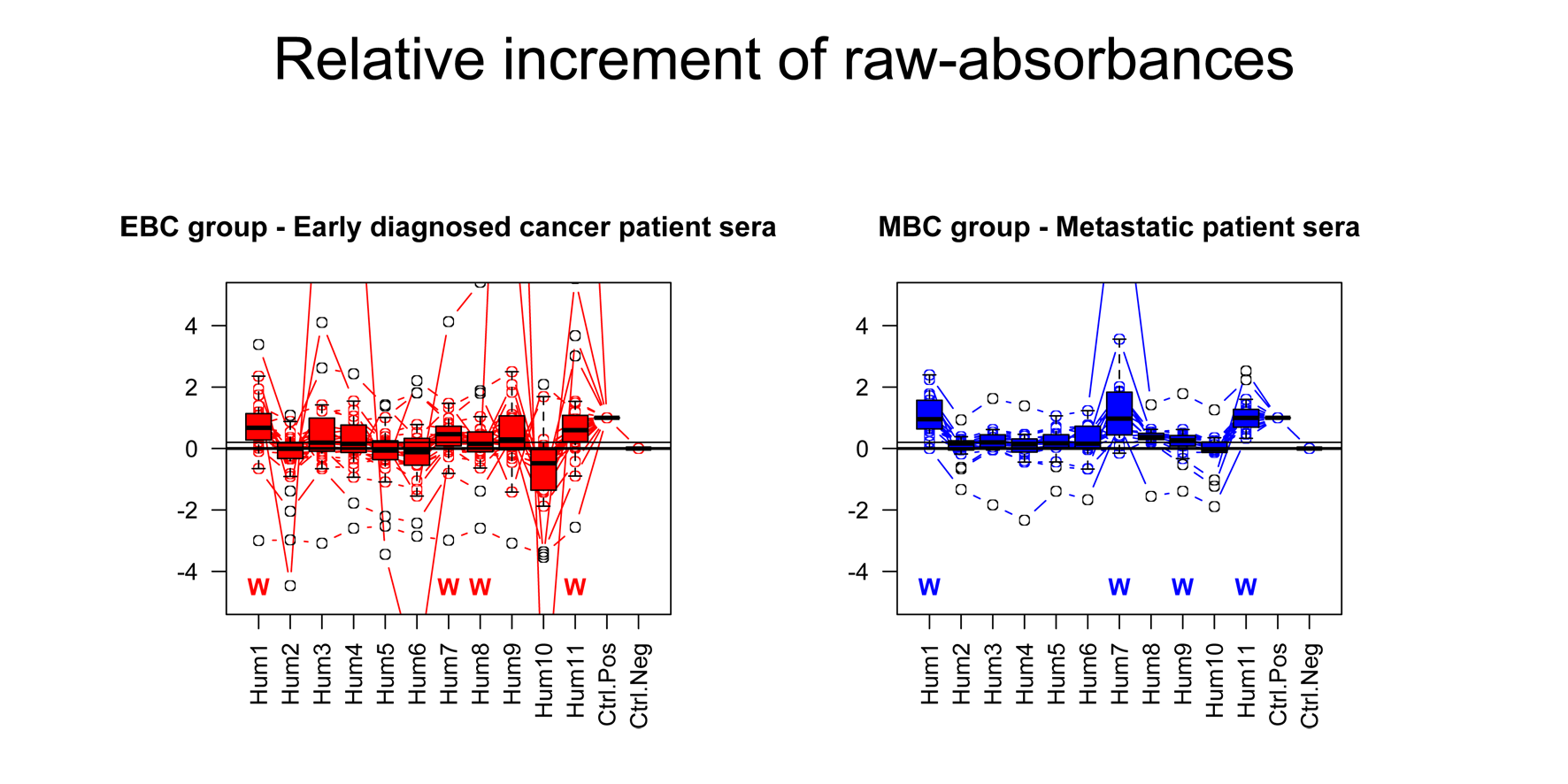

Supplement: Figure S7 — Human LFPD. Relative increment normalization of triplicates: raw absorbances of 60 ELISA experiments on human sera have been normalized by dividing each by the absorbance increment in the two control spots of each replicate. Normalized profile of 13 wells displayed by health status group: disease free (green), early-diagnosed cancer (red), metastatic (blue). (TIF) [file pone.0058358.s007.tif]

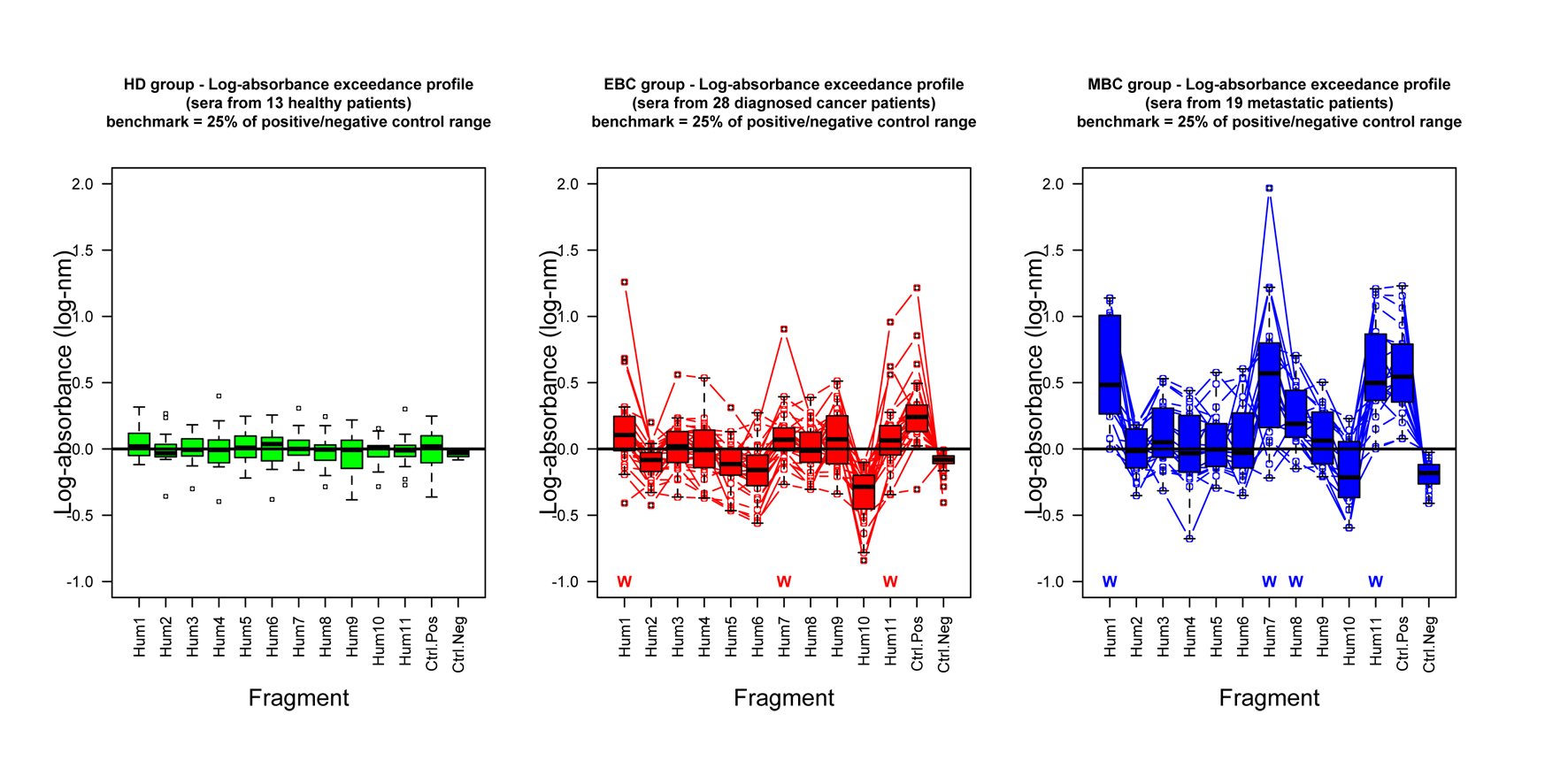

Supplement: Figure S8 — Human LFPD. Log-absorbance exceedance normalization of triplicates: log-absorbances of 60 ELISA experiments on human sera have been normalized by subtracting a 25% of the log-absorbance increment in the two control spots of each replicate. Normalized profile of 13 wells displayed by health status group: disease free (green), early-diagnosed cancer (red), metastatic (blue). Letters “W” (“T”) those epitopes for which a significant binding has been detected (p-value<0.05) with the Wilcoxon test (Student T test, whenever appropriate). (TIF) [file pone.0058358.s008.tif]

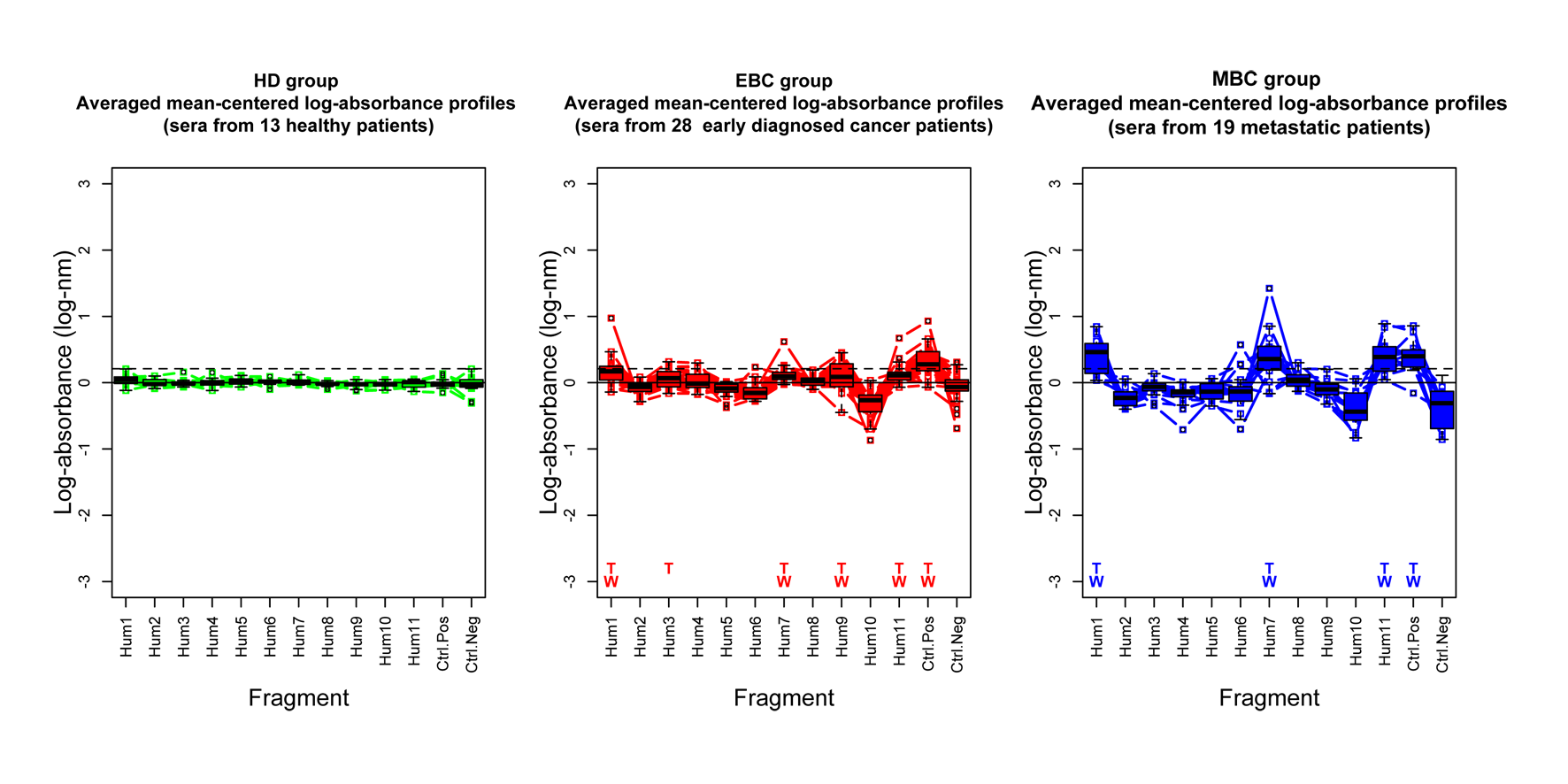

Supplement: Figure S9 — Human LFPD. Mean-centering normalization of triplicates. Same data as in Figure S4 with superimposed boxplots for each fragment to highlight summary characteristics of the overall profile shape. (TIF) [file pone.0058358.s009.tif]

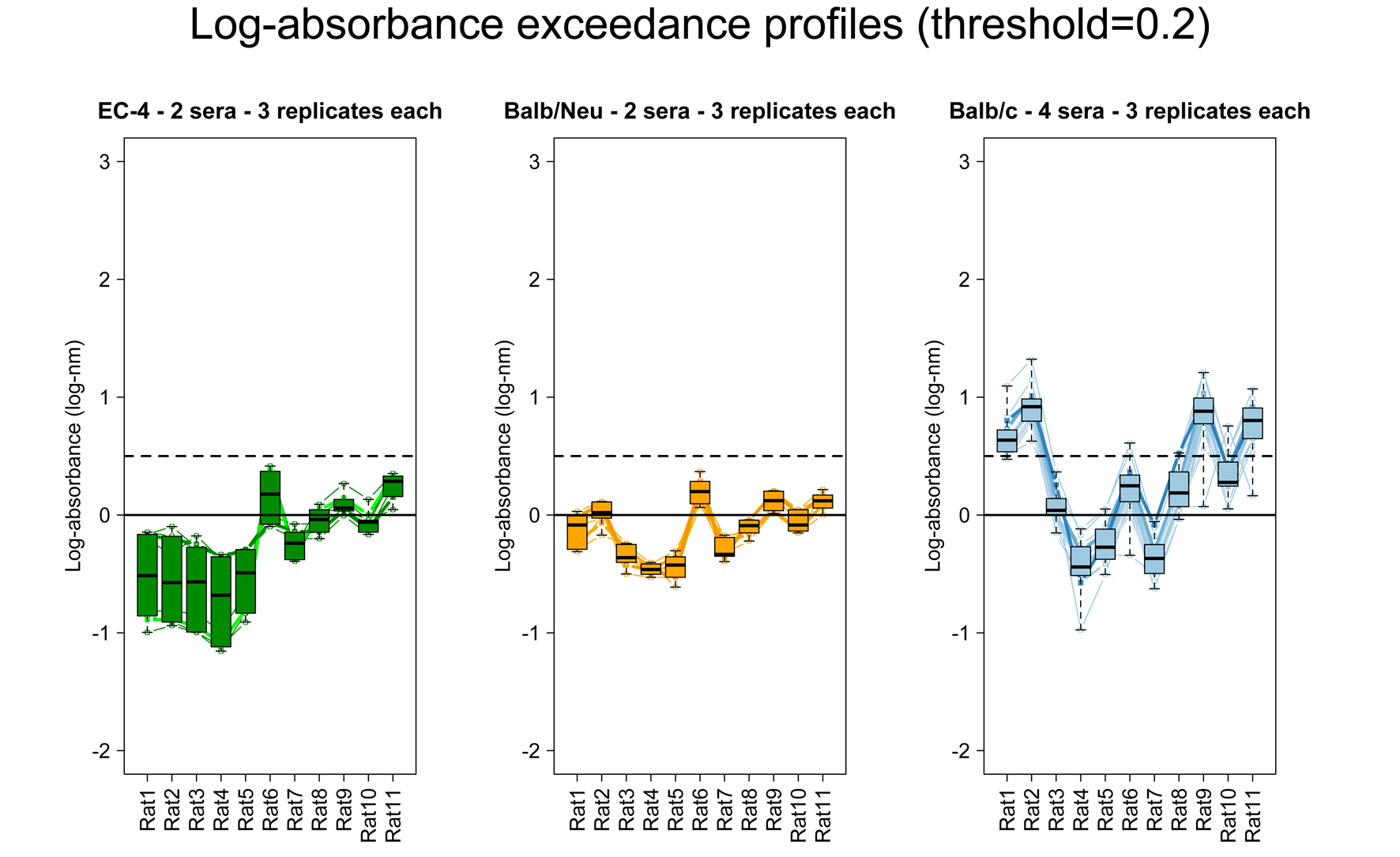

Supplement: Figure S10 — Rat LFPD. Relative increment normalized absorbance of 8 ELISA experiments (triplicated) on rat sera. Absorbance profile of 11 wells corresponding to 11 fragments (rat1-rat11). Results are displayed by mice type: EC4-TM vaccinated Balb/c (dark-green), EC-TM vaccinated tolerant transgenic BALB-neuT (orange), EC-TM vaccinated non tolerant wild-type Balb/c (cyan). (TIF) [file pone.0058358.s010.tif]

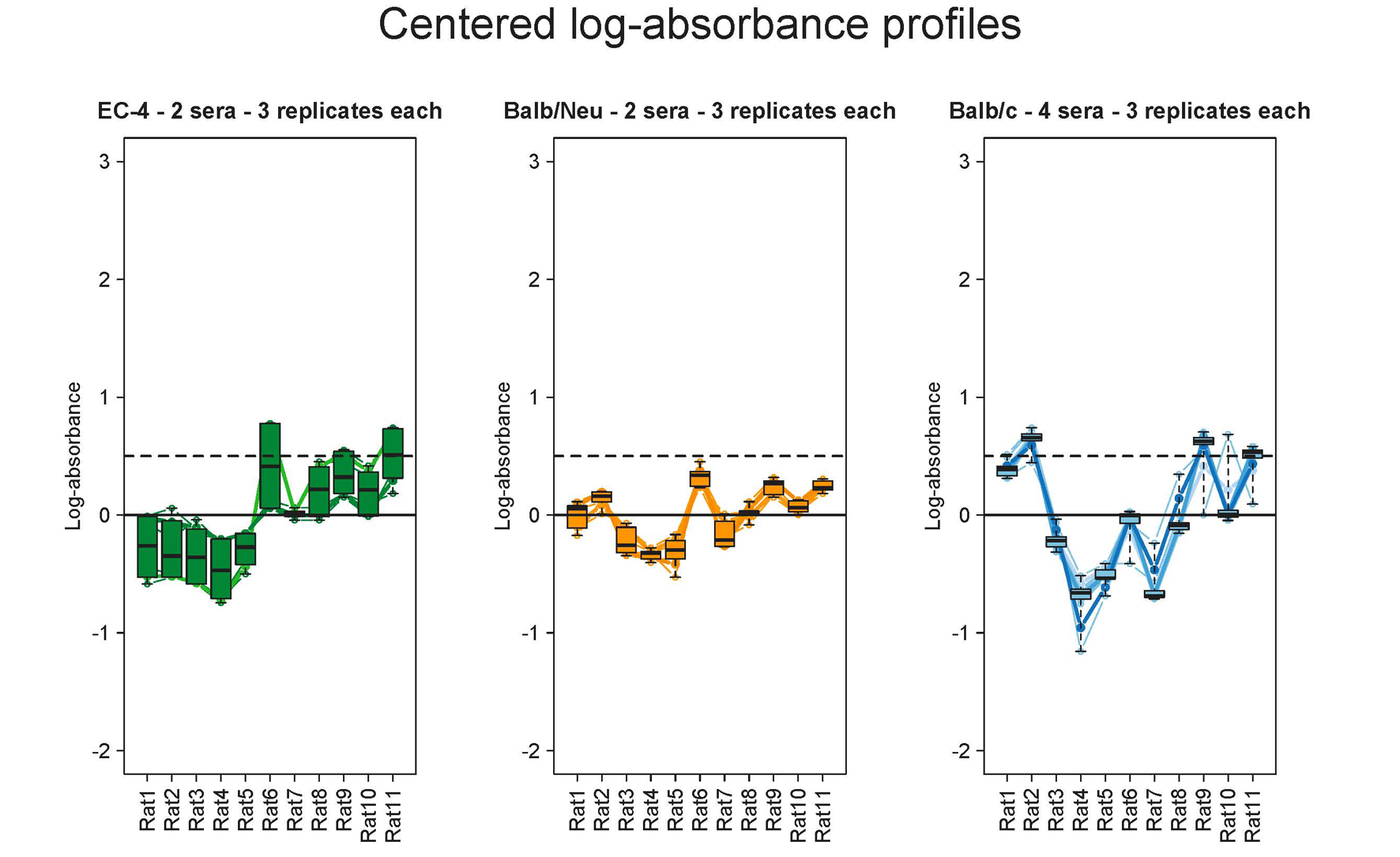

Supplement: Figure S11 — Rat LFPD. Mean centered normalized log-absorbance of 8 ELISA experiments (triplicated) on rat sera. Absorbance profile of 11 wells corresponding to 11 fragments (rat1-rat11). Results are displayed by mice type: EC4-TM vaccinated Balb/c (dark-green), EC-TM vaccinated tolerant transgenic BALB-neuT (orange), EC-TM vaccinated non tolerant wild-type Balb/c (cyan). (TIF) [file pone.0058358.s011.tif]

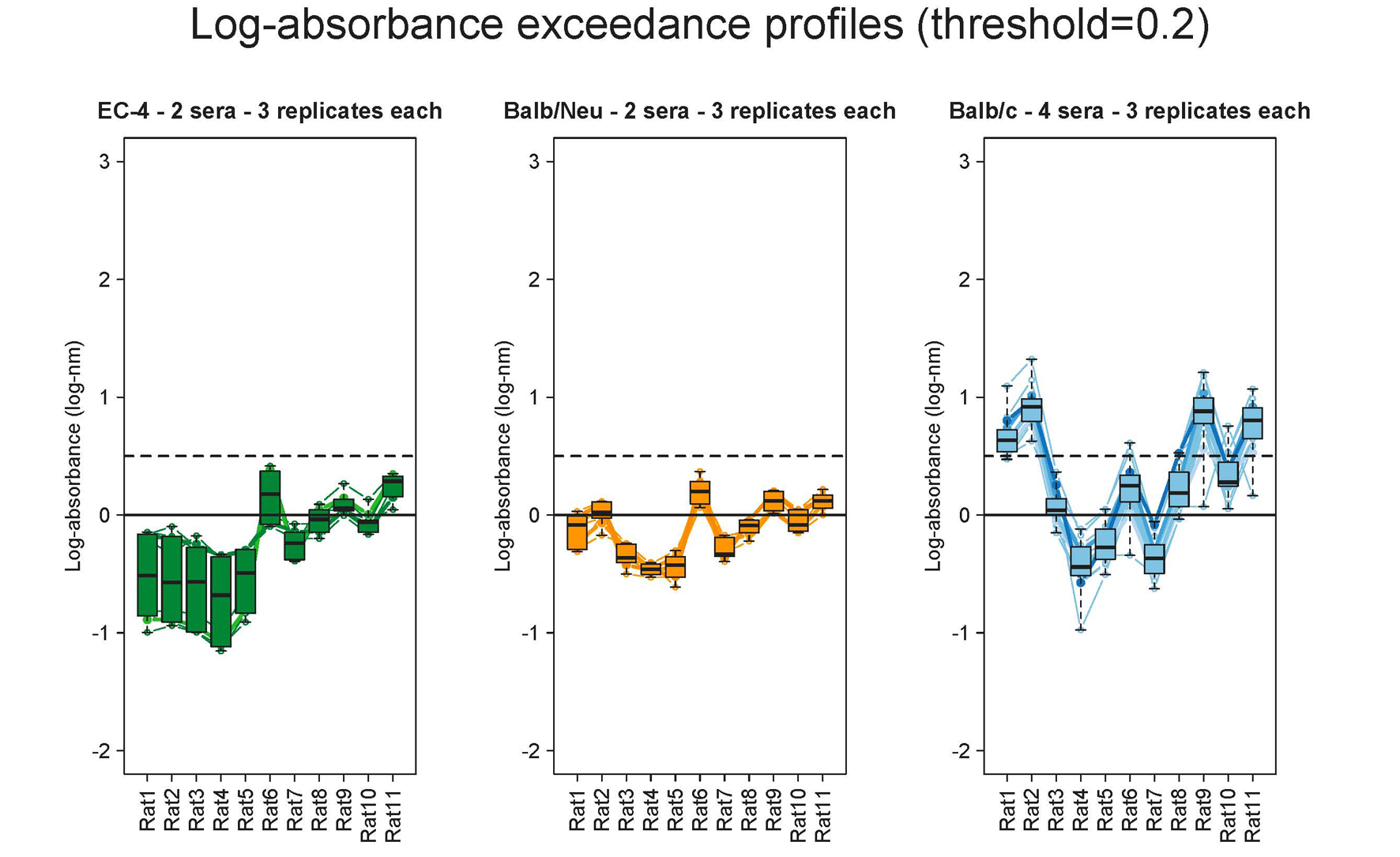

Supplement: Figure S12 — Rat LFPD. Log-absorbance exceedance normalization of 8 ELISA experiments (triplicated) on rat sera. Absorbance profile of 11 wells corresponding to 11 fragments (rat1-rat11). Results are displayed by mice type: EC4-TM vaccinated Balb/c (dark-green), EC-TM vaccinated tolerant transgenic BALB-neuT (orange), EC-TM vaccinated non tolerant wild-type Balb/c (cyan). (TIF) [file pone.0058358.s012.tif]

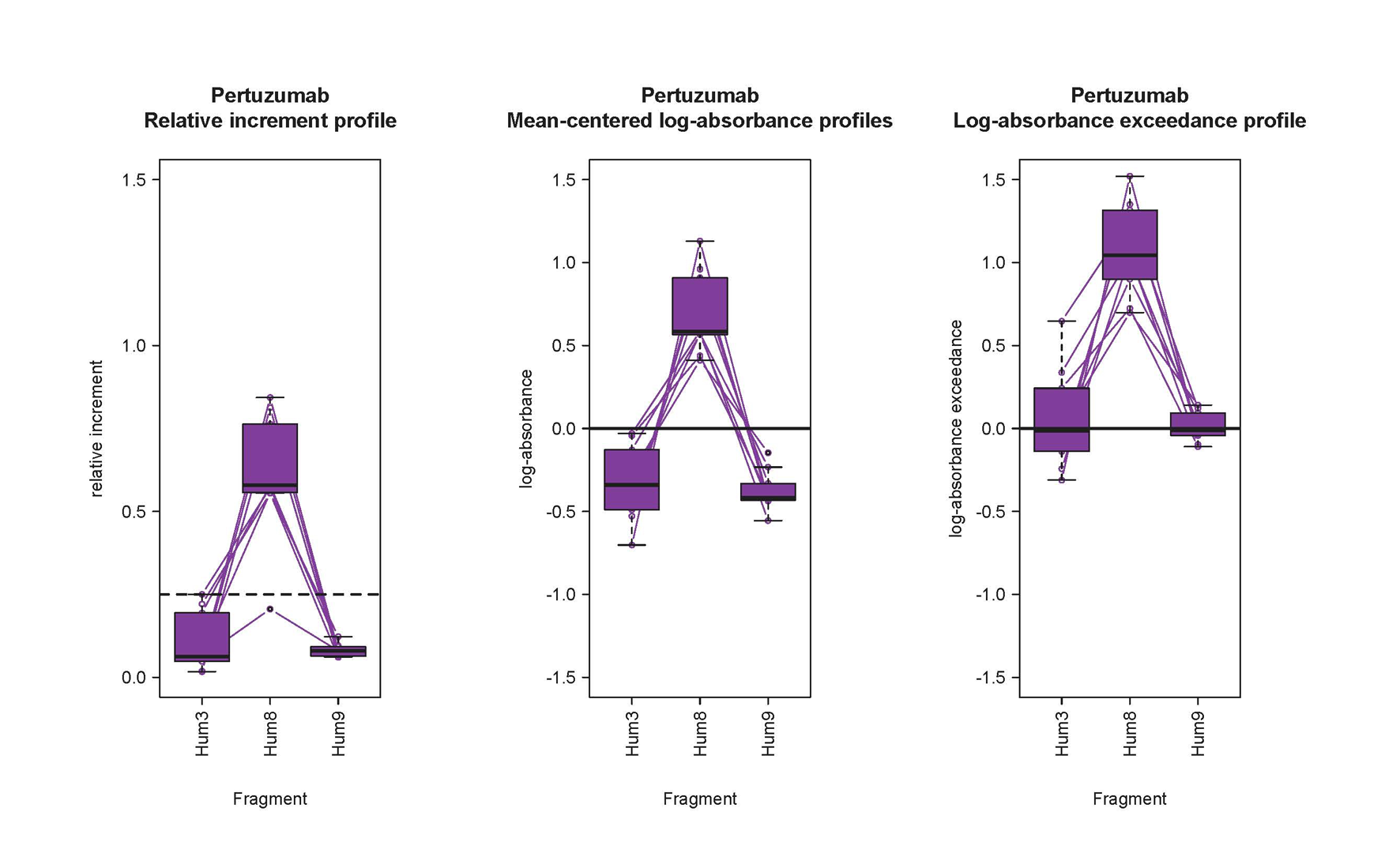

Supplement: Figure S13 — Human LFPD. Pertuzumab monoclonal antibody absorbance by alternative normalizations of triplicates: normalized absorbances of 3 ELISA experiments. Normalized profiles of 3 wells corresponding to fragments (hum3, hum8, hum9) displayed by normalization type. (TIF) [file pone.0058358.s013.tif]

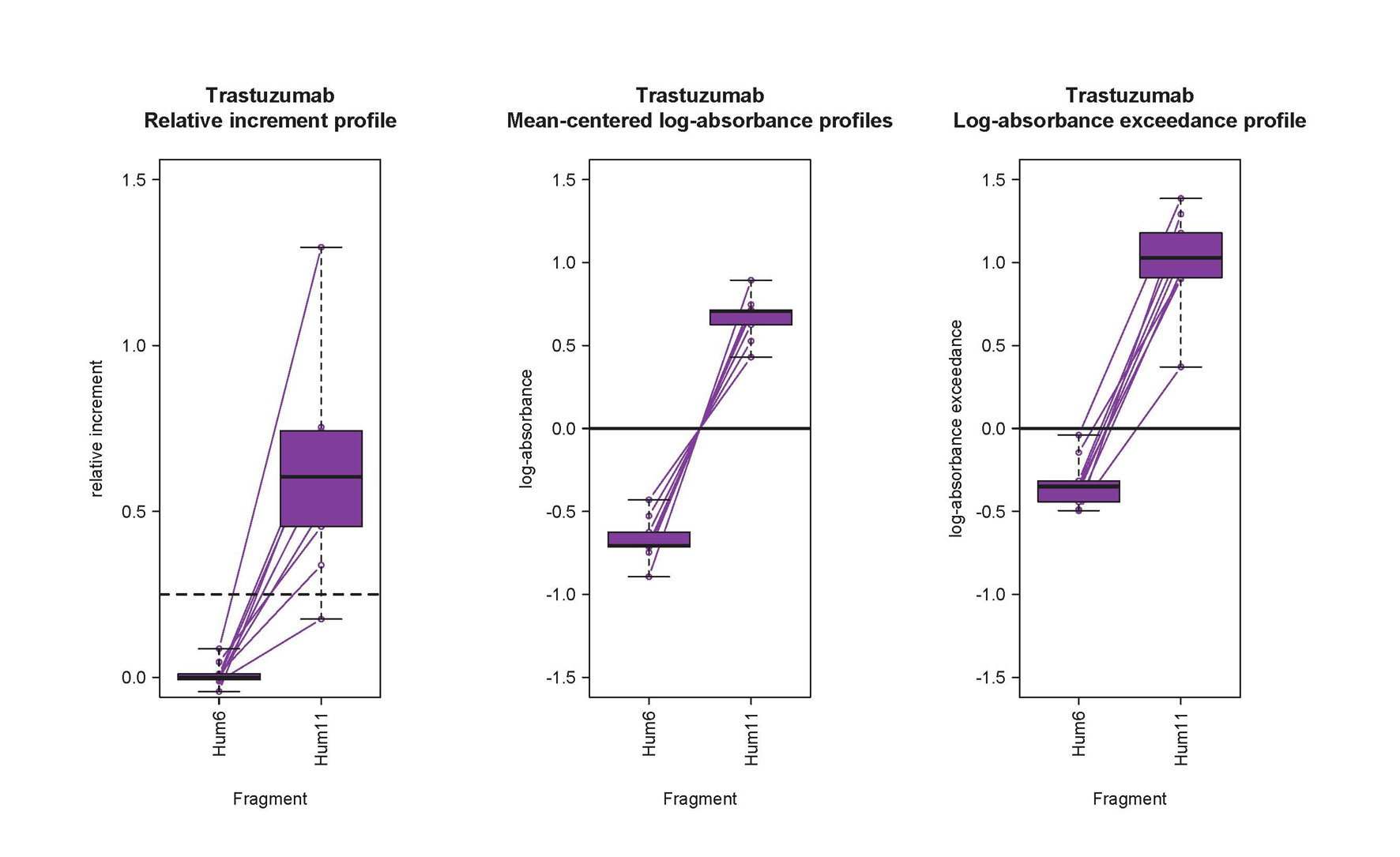

Supplement: Figure S14 — Human LFPD. Trastuzumab monoclonal antibody absorbance by alternative normalizations of triplicates: normalized absorbances of 3 ELISA experiments. Normalized profiles of 2 wells corresponding to fragments (hum6 and hum11) displayed by normalization type. (TIF) [file pone.0058358.s014.tif]

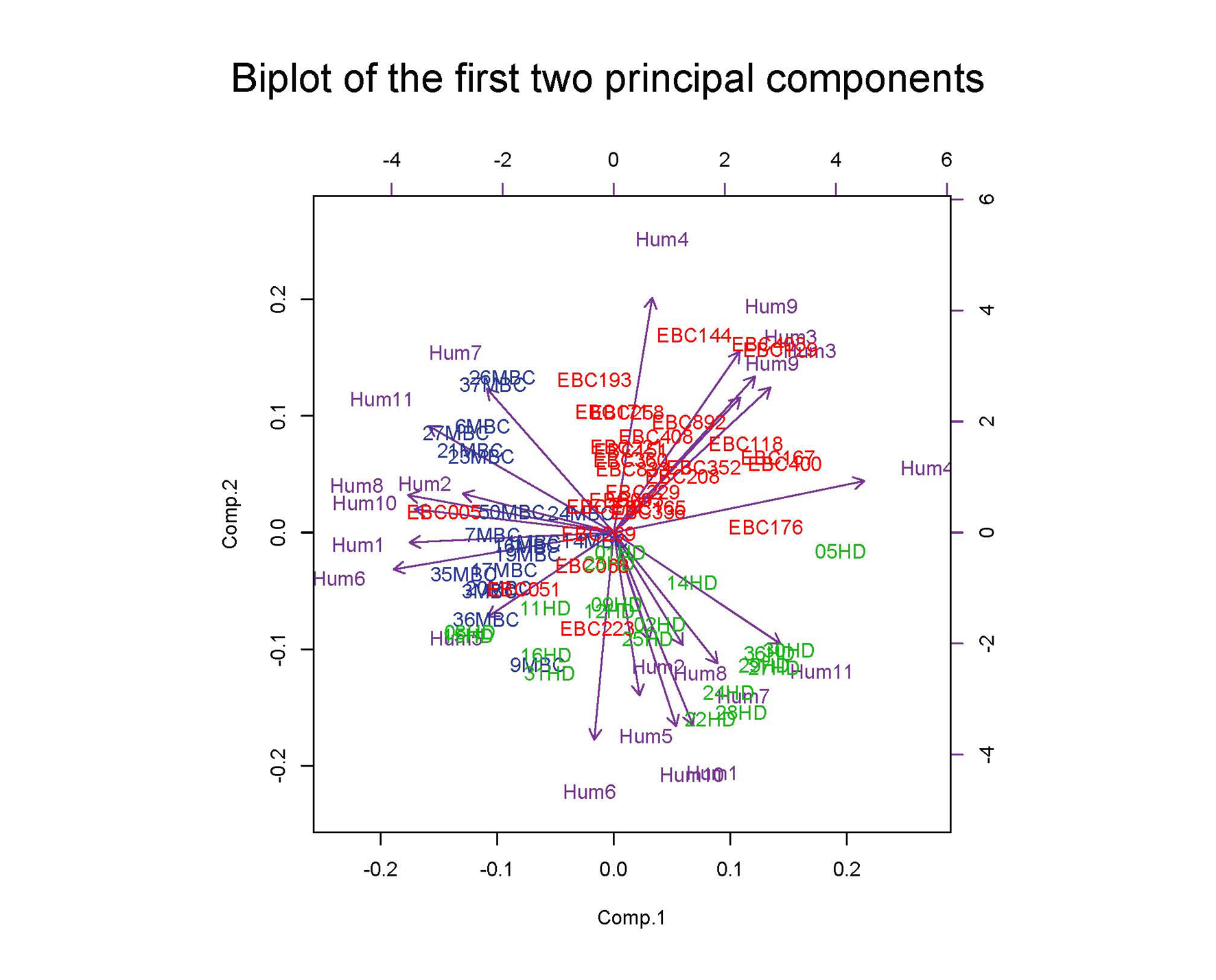

Supplement: Figure S15 — Human LFPD. Biplot of the first two principal components extracted from combining original normalized profiles and their ranks. (TIF) [file pone.0058358.s015.tif]

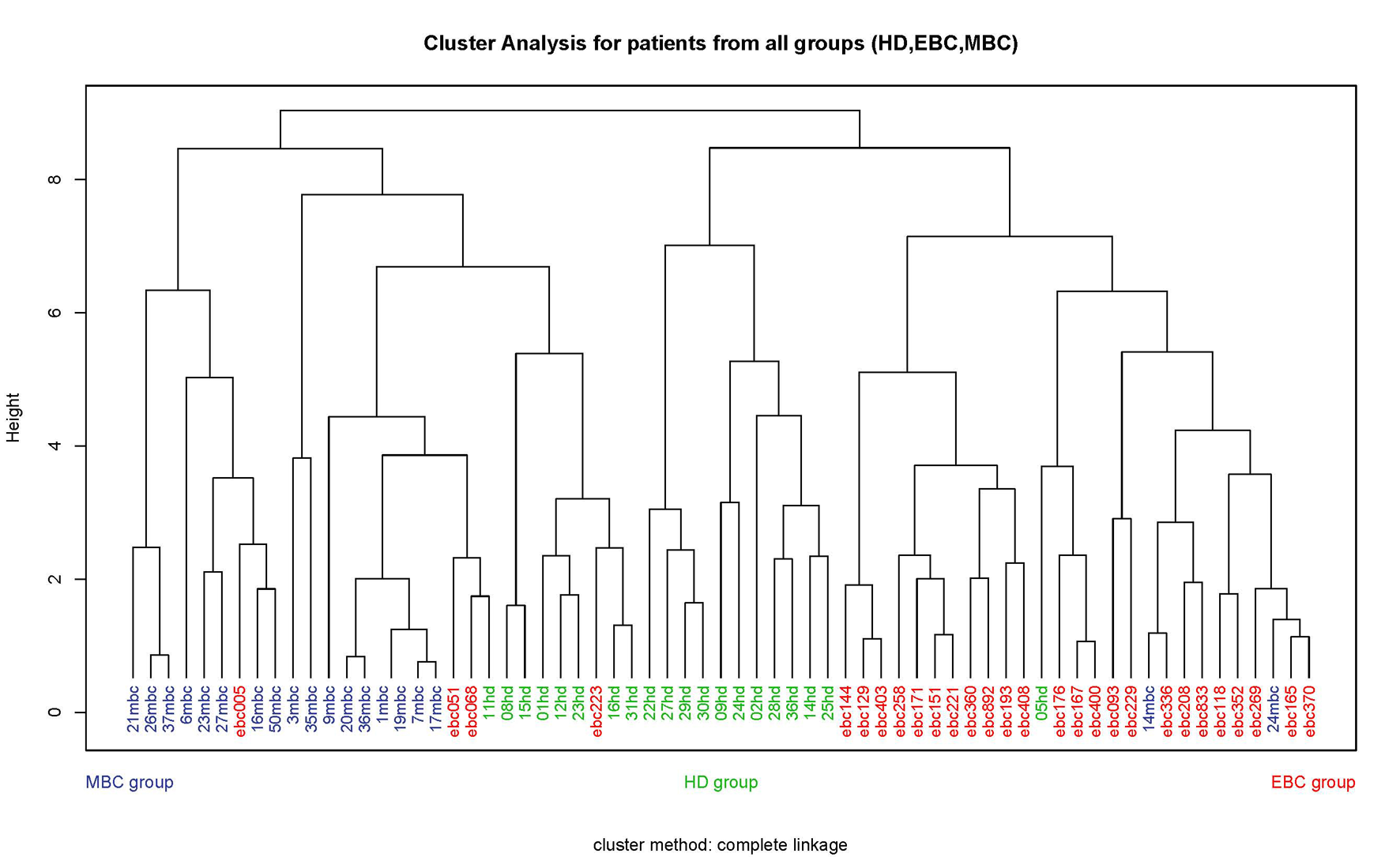

Supplement: Figure S16 — Human LFPD. Unsupervisioned hierarchical clustering obtained from mean centered log-absorbance. Inpt data were based on the first 6 principal components extracted from combining original normalized profiles and their ranks. (TIF) [file pone.0058358.s016.tif]

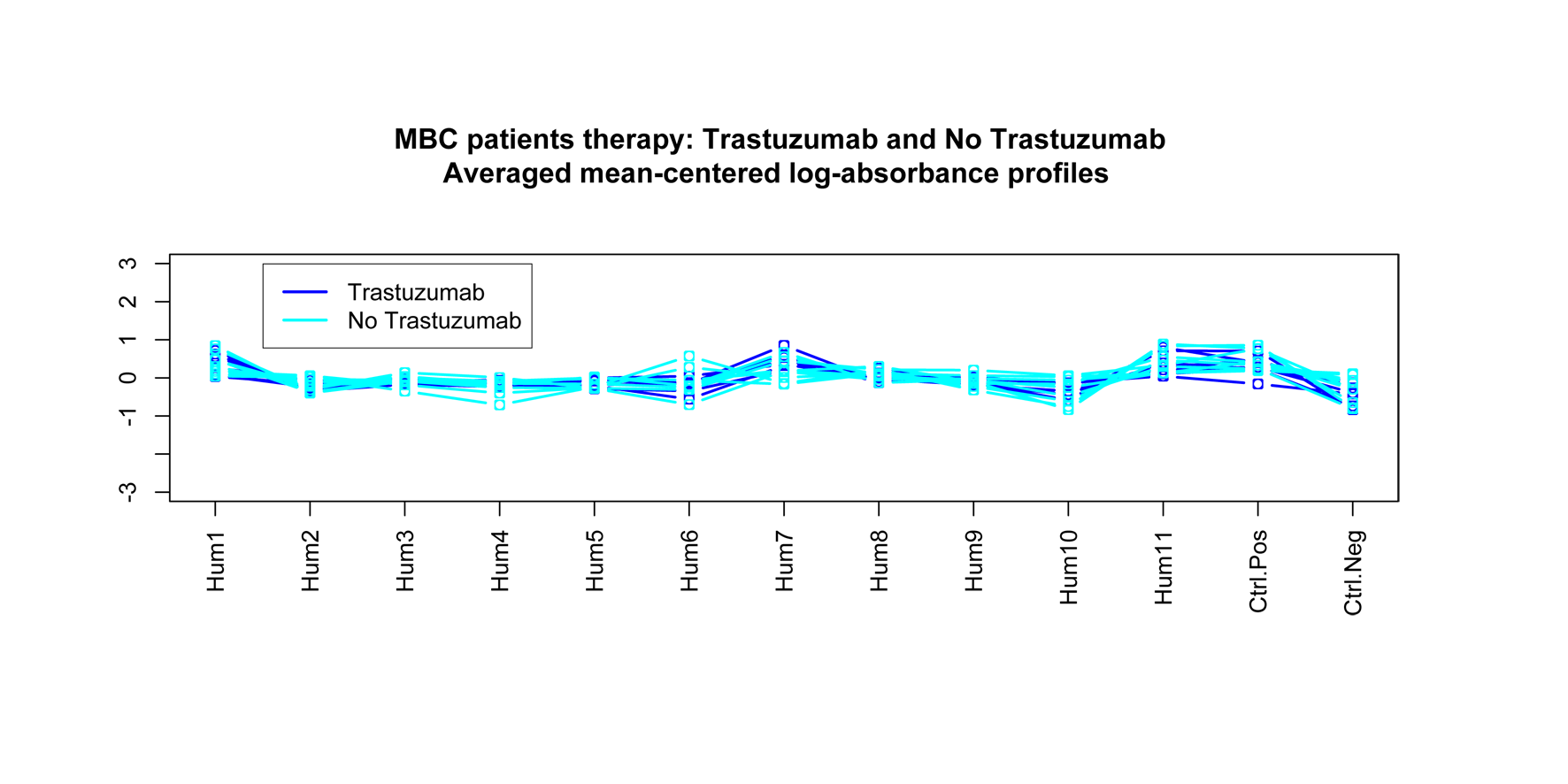

Supplement: Figure S17 — Raw absorbance of ELISA experiments (triplicated) on human sera of two groups of cancer patients: treated with Trastuzumab (light blue) not treated with Trastuzumab (dark blue). Absorbance profile of 13 wells corresponding to 11 fragments (hum1-hum11) and 2 control wells (CtrlPos, positive control = whole HER-b protein; CtrlNeg, negative control = phage). (TIF) [file pone.0058358.s017.tif]

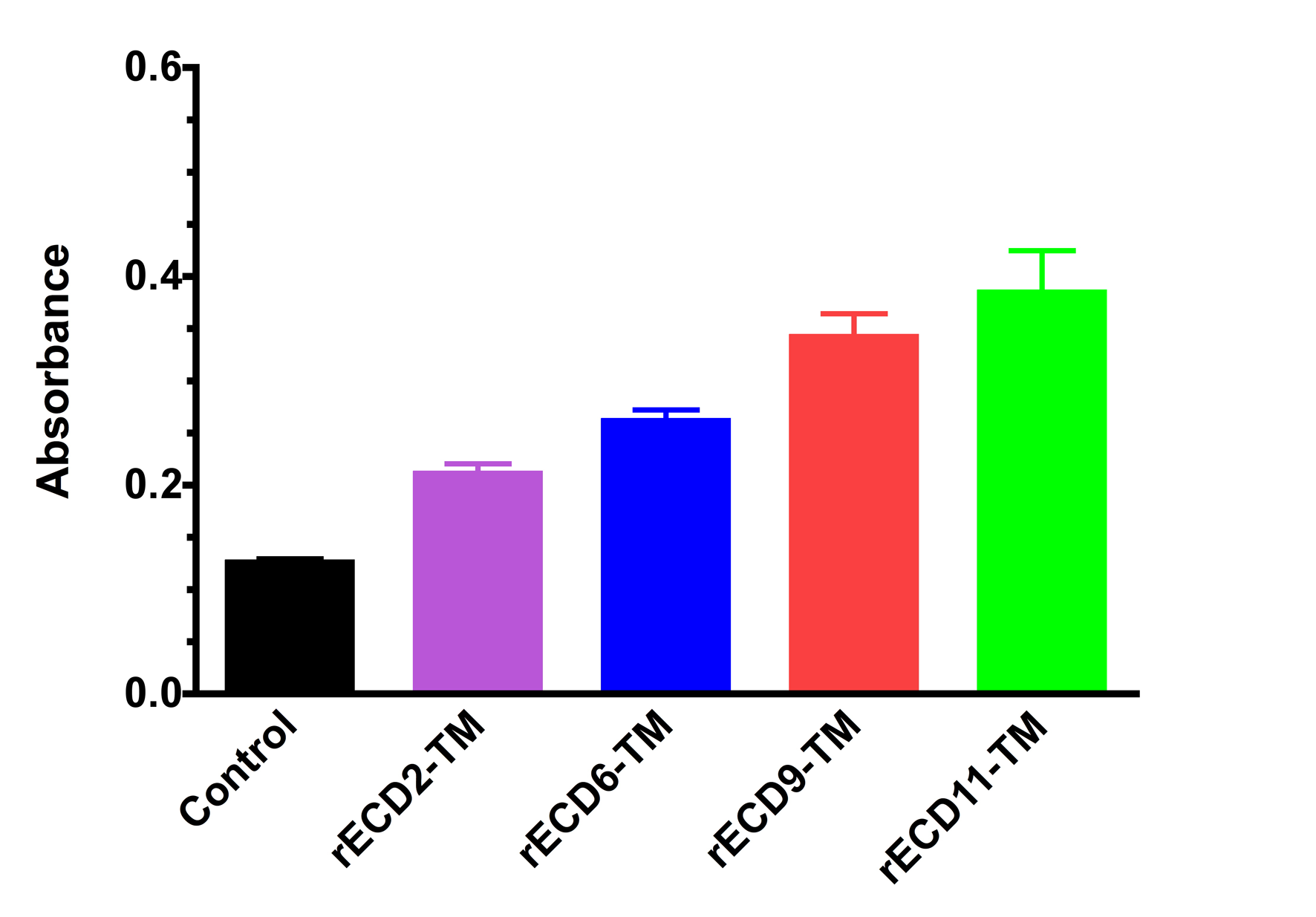

Supplement: Figure S18 — Induction of anti-HER2 antibody response by epitope-based vaccines. Balb/c mice were vaccinated with rECD2-TM, rECD6-TM, rECD9-TM, rECD11-TM. Binding of rat HER2 expressing 3T3/NKB cells with immune sera was measured by ELISA assay as described in Materials and Methods. Sera from mice vaccinated with pFuse-TM empty plasmid were used as negative control. Results are expressed as absorbance (optical density at 405 nm). Data are shown as mean ± SEM (n = 4). (TIF) [file pone.0058358.s018.tif]

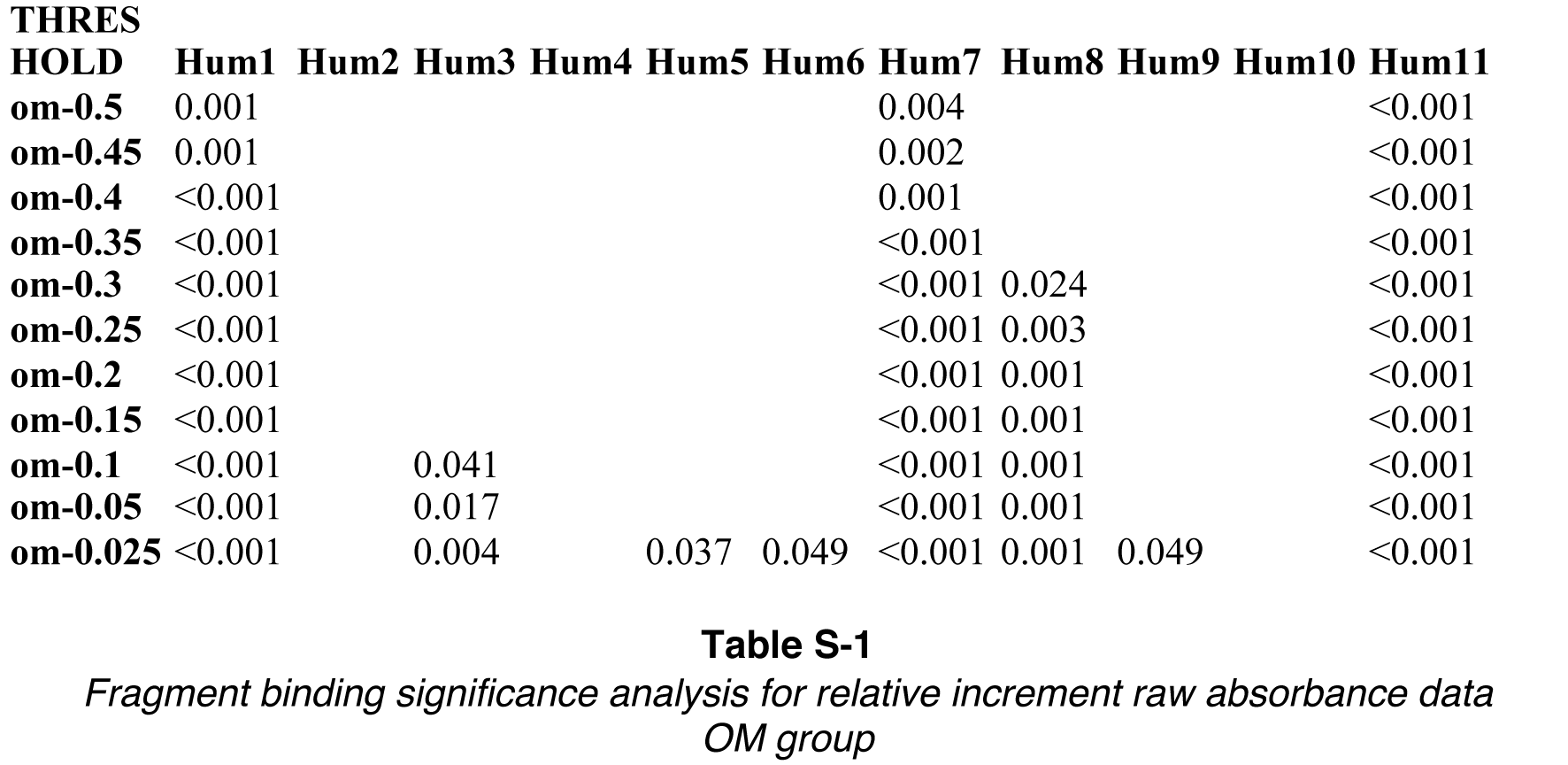

Supplement: Table S1 — Fragment binding significance analysis for relative increment raw absorbance data OM group (metastatic breast cancer patients). (TIF) [file pone.0058358.s019.tif]

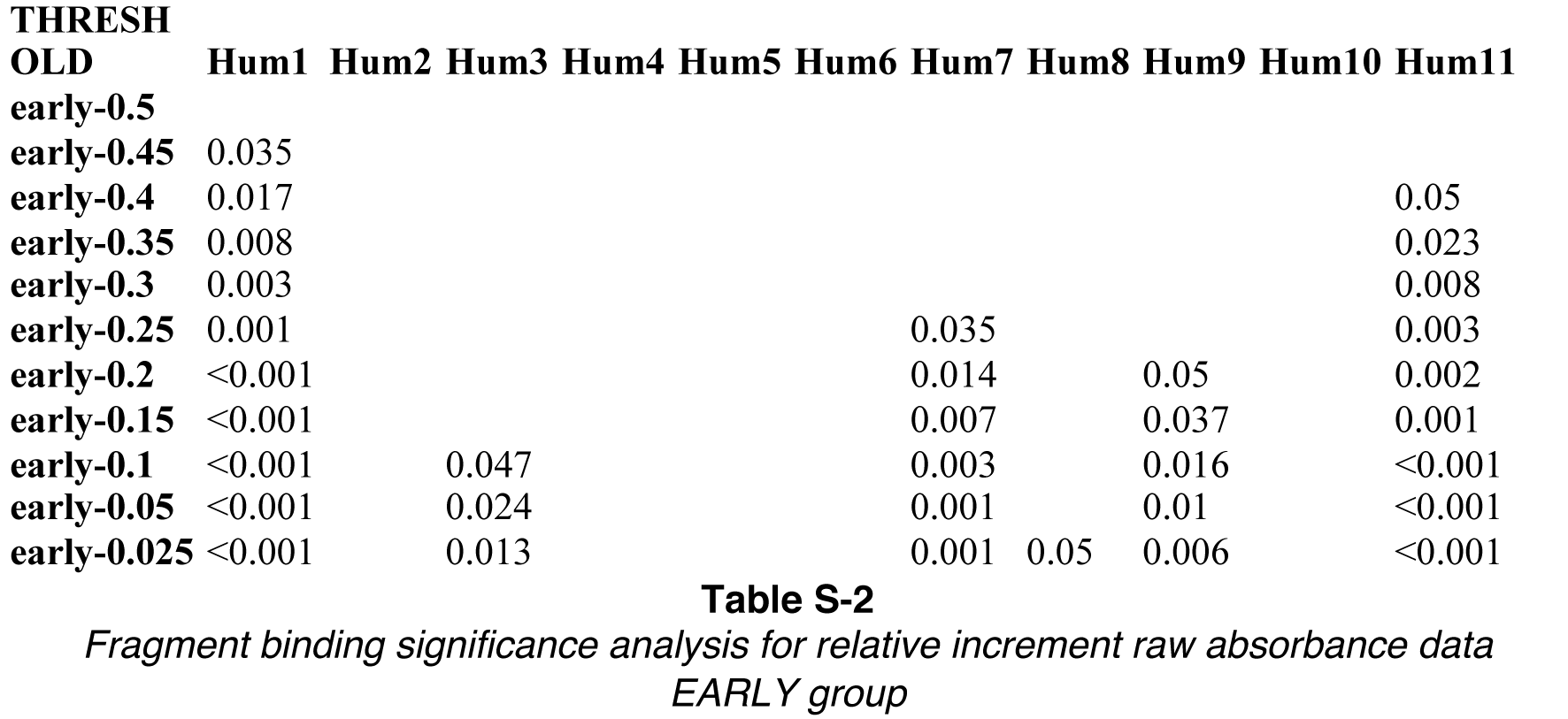

Supplement: Table S2 — Fragment binding significance analysis for relative increment raw absorbance data EARLY group (early breast cancer patients). (TIF) [file pone.0058358.s020.tif]

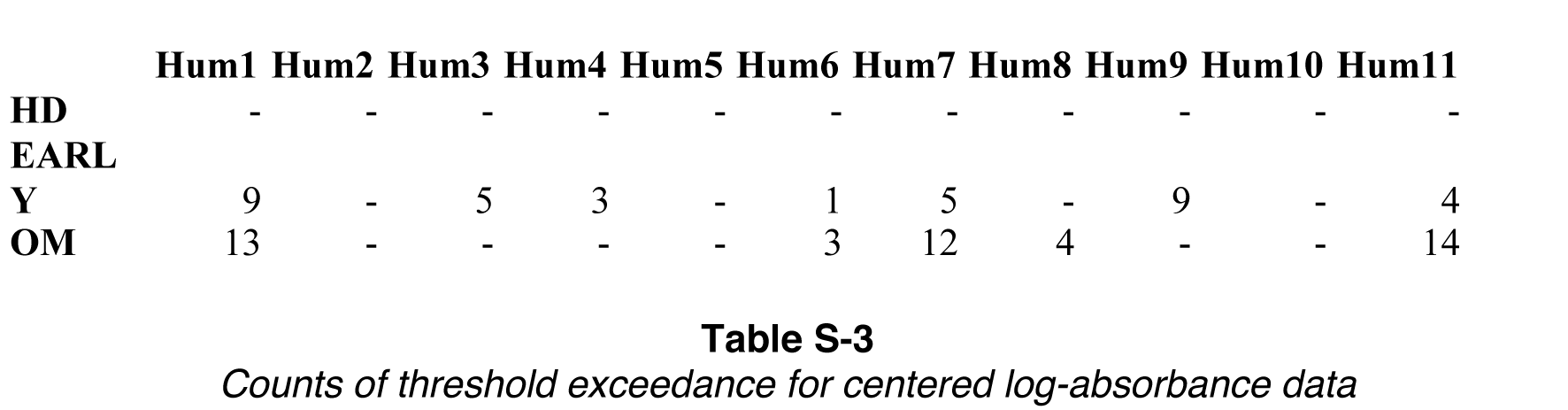

Supplement: Table S3 — Counts of threshold exceedance for centered log-absorbance data. (TIF) [file pone.0058358.s021.tif]

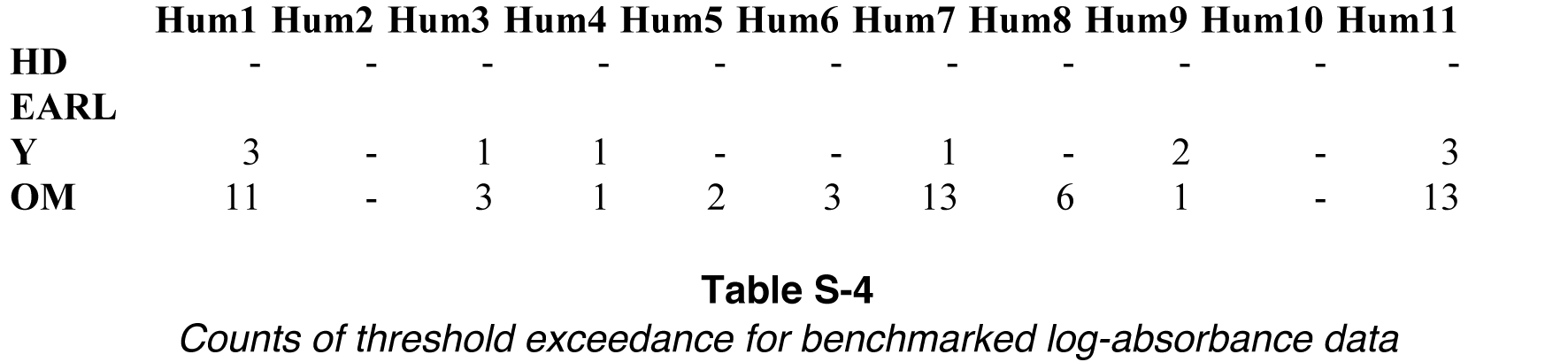

Supplement: Table S4 — Counts of threshold exceedance for benchmarked log-absorbance data. (TIF) [file pone.0058358.s022.tif]
