# Supplementary material for: Identification of Relevant Conformational Epitopes on the HER2 Oncoprotein by Using Large Fragment Phage Display (LFPD)
Source: PLoS One. 2013 Mar 28;8(3):e58358. doi: 10.1371/journal.pone.0058358 (PMC3610777; doi:10.1371/journal.pone.0058358)
Supplement: Methods S1 — Statistical analysis, including data transformation and normalization, testing significant overall epitope recognition, highlighting individual epitope recognition, hints of group discrimination by profile pattern. (DOC) [file pone.0058358.s023.doc]

**Methods S1**

**Statistical Analysis**

**Data transformation and normalization**

Main experimental quantitative outcome has been measured in terms of absorbance expressed in nm. Human sera has been used for 60 ELISA experiments corresponding to 13 healthy patients (HD), 28 early diagnosed cancer patients (EARLY) and 19 metastatic cancer cases (OM).

Each experiment yielded a technical triplicate corresponding to the same human sera.

Lines in Figure S-3 show raw data profiles of individual replicates. Each line consists of absorbance measurements of 11 wells (denoted on the x-axis with Hum1, Hum2, …., Hum13) corresponding to each LFPD fragment and 2 control wells corresponding, respectively, to negative control (Ctrl.Neg) with phages containing no protein fragment and positive control (Ctrl.Pos) with phages containing the whole protein. Log-transforming raw data is used as a normalization step and yields lines which are displayed in Figure S-4. The marginal distributions of binding signals as detected from the different wells are less asymmetric.

Figures S-5 and S-6 show that individual profiles are overall lower for HD sera, higher for the EARLY group and even higher for OM. Indeed replicate profiles from OM sera show similar non-steady pattern possibly confounded by the fact that each profile has its own “height”. This is also apparent for the EARLY group.

Indeed a proper re-centering of each profile with the subtraction of the arithmetic mean of log-absorbance measured over Hum1 … Hum11 reveals a more coherent pattern (see Figure S-5) and a final averaging of centered log-absorbances over technical triplicates are displayed in Figure S-6.

From the above exploratory description it is apparent the individual heterogeneity of each profile is systematic and this suggests that there can be differences in the number and the selection of epitopes which are recognized from each individual serum/patient.

Hence we will investigate two different aspects of epitope binding:

- overall significant evidence of binding for each fragment
- individual specific subsets of bound epitopes.

**Testing significant overall epitope recognition**

Due to individual heterogeneity the original raw quantitative outcome must be properly processed in order to get an appropriate evidence of epitope binding.

We have set-up alternative quantitative methodologies to assess whether or not there is evidence of epitope binding overall for each fragment.

The first one is based on a very easy-to-interpret relative increment of raw absorbance. The difference between the absorbance corresponding to a single LFPD fragment and the absorbance of the negative control is divided by the absorbance difference between positive and negative controls in formula

rel.incr=(raw.abs- Ctrl.Neg.abs)/(Ctrl.Pos.raw.abs-Ctrl.Neg.raw.abs)

Such increments are averaged over triplicates and the overall distributions corresponding to all fragments are displayed in Figure S-7. There is some evidence of wild fluctuation of individual profiles but an appropriate non parametric test to verify whether the overall median increment is significantly larger than a suitably elicited threshold R gives the Table S-1 and Table S-2 of p-values for the OM and EARLY groups.

Choosing R in the range 0.15-0.2 yields an overall statistical evidence (p-value <0.05) of epitope binding for Hum1, Hum7, Hum8, Hum11 for the OM group and Hum1, Hum7, Hum9, Hum11 for the EARLY group.

An alternative quantification and statistical significance analysis can be based on the log-absorbance increment that is obtained comparing each fragment/well signal with respect to the signals that is obtained with both positive and negative control. It seems reasonable to subtract frow the log-absorbance a *benchmark* quantity which is obtained adding to the negative control signal a fraction – say *B* – of the range obtained considering the postive and negative control wells. In formula

bench = Ctrl.Neg.log.abs+ *B* * **range**(Ctrl.Neg.log.abs,Ctrl.Pos.log.abs)

This corresponding differences are displayed in Figure S-8 where averaged triplicate profiles have been superimposed with each fragment marginal boxplot.

The T/W letters on the bottom of the graph highligth those wells/fragments for which an appropriate statistical test (W=wilcoxon, T=Student T) gives a p-value less than 0.05 for testing whether the increment is significantly greater than 0.05.

Choosing a benchmark fraction *B*=0.25 and a parametric T-test one can conclude that fragments Hum1, Hum3, Hum7, Hum8, Hum11 are overall significantly bound by Abs present in metastatic patient sera while fragments Hum1, Hum7, Hum9, Hum11 are overall significantly bound by Abs present in early diagnosed patient sera. No evidence of significant binding is gathered from repeating the tests with the HD group measurements. Indeed the ad-hoc calibration B=0.25 well matches with the fact that with this choice the standardized absorbances of the different fragments in the HD group (see first left panel in Figure S-8) are well centered around 0.

Finally, we have considered as a quantitative description of epitope binding the centered profiles as from Figure S-9. Differently from the previous quantification this mean centered profile does not take into account the negative and positive control spots hence its interpretation is less neat although profile behaviour is more consistent and stable within each group. Again one can see that there is evidence of significant binding (greater than a minimal positive threshold of 0.01 in terms of centered log-absorbance) for fragments Hum1, Hum7, Hum11 in OM group and Hum1, Hum7, Hum9, Hum11 in the EARLY group.

Even with this quantification no overall evidence of significant binding is gathered from repeating the tests for the HD group.

**Highlighting individual epitope recognition**

If we consider the centered profiles of log-absorbance one can take advantage of the evidence from the healthy patient group to calibrate a minimal threshold of log-absorbance for which no sera in the HD group has a centered log-absorbance in correspondence to any fragment which exceeds that threshold. Hence we take as a reference threshold L=0.21 and we state that the Abs present in a single sera show evidence of bound epitope whenever the centered log-absorbance exceeds L. From this definition it follows that 18 sera out of the 28 sera in the EARLY group (64%) recognize at least one fragment/epitope, while there are 18 sera out of the 19 from the OM group (94%) which recognize at least one fragment/epitope. Table S-3 shows the number of sera which exceed the given threshold L=0.21 for each fragment. This table confirms that in the EARLY group the main bound epitopes correspond to fragments Hum1 Hum7 and Hum9 and Hum11 with some more evidence of binding for Hum3 which is detected as frequently as Hum7 and Hum11. Similarly, the table confirms that in the OM group the most frequently bound epitopes correspond to Hum1 Hum7 and Hum11.

We have tried to consider a similar strategy for highlighting individual epitope recognition using the other two quantifications which have been used for highlighting overall epitope binding. Unfortunately the other measures of standardized and relative increments do show a much wilder individual behavior and the resulting tables are then less evident than the previous Table S-1. More precisely for benchmarked differences of log-absorbance we report in Table S-2 the same counts of threshold exceedance where a threshold L=0.4 has been elicited from the HD group as previously done for centered log-absorbances. No sensible threshold could be elicited for the relative increments of raw absorbance (Table S-4).

**Hints of group discrimination by profile pattern**

We have also used the quantitative outcomes in the different normalized versions to verify whether different groups can be characterized by specific absorbance profile. After trying the direct use of the quantitative outcome profile for each patient as a base for discrimination we have eventually found that rather the internal ordering of the absorbance outcome has some interesting potential for group discrimination. Figure S-15 shows the first two principal components based on an appropriate combined use of absorbance orderings. Figure S-16 shows the dendrogram obtained from hierarchical clustering based on the first 6 principal components. In both cases the group discrimination is quite evident. More investigation with larger groups of patients can statistically validate this preliminary descriptive findings.
